# Comparison of the chloroplast peroxidase system in the chlorophyte *Chlamydomonas reinhardtii*, the bryophyte *Physcomitrella patens*, the lycophyte *Selaginella moellendorffii *and the seed plant *Arabidopsis thaliana*

**DOI:** 10.1186/1471-2229-10-133

**Published:** 2010-06-28

**Authors:** Nicola T Pitsch, Benjamin Witsch, Margarete Baier

**Affiliations:** 1 Plant Science Institute, Heinrich-Heine-University, Universitätsstraße 1, 40225 Düsseldorf, Germany; 2 Plant Physiology, Freie Universität Berlin, Königin-Luise-Straße 12-16, 14195 Berlin, Germany

## Abstract

**Background:**

Oxygenic photosynthesis is accompanied by the formation of reactive oxygen species (ROS), which damage proteins, lipids, DNA and finally limit plant yield. The enzymes of the chloroplast antioxidant system are exclusively nuclear encoded. During evolution, plastid and mitochondrial genes were post-endosymbiotically transferred to the nucleus, adapted for eukaryotic gene expression and post-translational protein targeting and supplemented with genes of eukaryotic origin.

**Results:**

Here, the genomes of the green alga *Chlamydomonas reinhardtii*, the moss *Physcomitrella patens*, the lycophyte *Selaginella moellendorffii *and the seed plant *Arabidopsis thaliana *were screened for ORFs encoding chloroplast peroxidases. The identified genes were compared for their amino acid sequence similarities and gene structures. Stromal and thylakoid-bound ascorbate peroxidases (APx) share common splice sites demonstrating that they evolved from a common ancestral gene. In contrast to most cormophytes, our results predict that chloroplast APx activity is restricted to the stroma in Chlamydomonas and to thylakoids in Physcomitrella. The moss gene is of retrotransposonal origin.

The exon-intron-structures of 2CP genes differ between chlorophytes and streptophytes indicating an independent evolution. According to amino acid sequence characteristics only the A-isoform of Chlamydomonas 2CP may be functionally equivalent to streptophyte 2CP, while the weakly expressed B- and C-isoforms show chlorophyte specific surfaces and amino acid sequence characteristics. The amino acid sequences of chloroplast PrxII are widely conserved between the investigated species. In the analyzed streptophytes, the genes are unspliced, but accumulated four introns in Chlamydomonas. A conserved splice site indicates also a common origin of chlorobiont PrxQ.

The similarity of splice sites also demonstrates that streptophyte glutathione peroxidases (GPx) are of common origin. Besides a less related cysteine-type GPx, Chlamydomonas encodes two selenocysteine-type GPx. The latter were lost prior or during streptophyte evolution.

**Conclusion:**

Throughout plant evolution, there was a strong selective pressure on maintaining the activity of all three investigated types of peroxidases in chloroplasts. APx evolved from a gene, which dates back to times before differentiation of chlorobionts into chlorophytes and streptophytes, while Prx and presumably also GPx gene patterns may have evolved independently in the streptophyte and chlorophyte branches.

## Background

Oxygenic photosynthesis leads to the formation of reactive oxygen species (ROS), such as singlet oxygen (^1^O_2_) and superoxide anions (O_2_^-^) [1]. The latter are rapidly converted to hydrogen peroxide (H_2_O_2_) [2]. H_2_O_2 _is scavenged by low molecular weight antioxidants, such as ascorbate and glutathione [1]. More efficiently, it is enzymatically inactivated by peroxidases [2-4]. Inside chloroplasts, the main peroxidases are ascorbate peroxidases (APx), peroxiredoxins (Prx) and glutathione peroxidases (GPx) [2-5].

Cyanobacteria are the closest relatives of the prokaryotic evolutionary ancestors of plastids. Like chloroplasts, they already protected themselves against the photooxygenic pressure of photosynthesis by the activity of APx, Prx and GPx [3,6-10]. In addition, many cyanobacteria encode a bifunctional ascorbate peroxidase-catalase (APx-Cat), which was not maintained in plants.

In seed plants, APx, GPx and Prx are the major plastid H_2_O_2 _scavenging enzymes [2,11,12]: APx reduce H_2_O_2 _at the thylakoid membrane and in the chloroplast stroma on the expense of ascorbate [11]. In parallel, GPx [12] and three types of peroxiredoxins, namely 2-Cys peroxiredoxins (2CP), peroxiredoxins Q (PrxQ) and type-II peroxiredoxins (PrxII) [13,14], protect chloroplasts against H_2_O_2 _and alkyl hydroperoxides via thiol-mediated reaction mechanisms [15-17]: 2CP, PrxII and GPx are stromal proteins [12-14,18], while PrxQ are targeted to the thylakoid lumen [19].

During plant evolution, the photosynthetic endosymbiont lost all its genes for antioxidant enzymes from its genome. Prior and during this process, the antioxidant enzymes were replaced by nuclear encoded homologs. The gene products are post-translationally targeted to chloroplasts [13,20,21]. The targeting signal is encoded in a N-terminal transit peptide [20-22]. The chloroplast isoforms of APx, GPx and PrxII have cytosolic counterparts [4,13,17,23], while in higher plants 2CP and PrxQ are exclusively found in chloroplasts [19,21]. Plant GPx show a high sequence similarity to animal GPx4, suggesting an eukaryotic origin [17]. For GPx of Arabidopsis, rice and barley, Margis et al. [17] postulated common origin of the genes encoding plastid and extra-plastidic isoforms. Paralogs evolved by gene duplication and subsequent gene-specific mutations. According to Margis et al. [17], the initial duplication took place at least before the emergence of gymnosperms.

In contrast, 2CP have been postulated to be of endosymbiotic origin [21]. In general, it is very likely that the chloroplast antioxidant protection system was arranged by combination of genes inherited by the heterotrophic eukaryotic ancestor cell of plants and of organellar origin. During more than one billion years of evolution, plants have faced strong environmental changes, such as long- and short-term temperature and light changes and variation of water, CO_2 _and O_2 _availability. The variable environment has challenged and shaped the antioxidant defense systems via influencing photosynthesis and photooxidative ROS formation.

Here, the chloroplast peroxidase system was compared in four model organisms, for which (almost) full genome information is available. The selected species represent different steps in chlorobiont evolution. We screened EST and genomic databases of the unicellular green alga *Chlamydomonas reinhardtii *(Volvocales, Chlorophyta), the moss *Physcomitrella patens *(Funariales, Bryophytina, Streptophyta) and the spike moss *Selaginella moellendorffii *(Selaginellales, Lycopodiophytina, Streptophyta) for open reading frames (ORFs) encoding chloroplast targeted APx, GPx and Prx.

Chlamydomonas belongs to the group of Chlorophyceae within the chlorophyte branch of Chlorobionta. The unicellular alga with one large chloroplast per cell, still forms phycoplasts instead of phragmoplasts upon cell division [24]. In the streptophyte branch, mosses, such as the ancestors of Physcomitrella, evolved approximately 500 million years ago and separated from cormophytes [25]. Within the cormophytes, the spike mosses diverted from the lineage of seed plant precursors approximately 400 million years ago [26].

Based on genome comparisons it will be shown that there has been a permanent selective pressure on the maintenance of APx, GPx and Prx activity in chloroplasts despite changes in the suborganellar distribution and in the gene copy number. Comparison of gene structures will indicate common or independent origins.

## Results

Genome resources at JGI http://www.jgi.doe.gov, TAIR http://www.arabidopsis.org, Cosmoss http://www.cosmoss.org and ChlamyDB http://www.chlamy.org provide automatically generated and annotated gene models for *Chlamydomonas reinhardtii, Physcomitrella patens, Selaginella moellendorffii *and *Arabidopsis thaliana *based on bioinformatic gene-predictions and improvement by BLAST-comparison of genomic DNA with EST data resources [27]. The automated approaches have two disadvantages: Firstly, if the sequence similarity of genes is high, the same ESTs can align to all genes and pretend expressional activity of various similar genes, even if only one gene is active. Secondly, due to the low sequence similarity of transit peptides even between related organisms, organelle targeting signals and terminal membrane anchors are often not recognized [28]. Sequence analysis then easily results in wrong predictions for protein localization.

In this study we aimed at the genome-wide identification and annotation of genes encoding chloroplast peroxidases, namely APx, GPx and Prx, in distantly related model organisms. The chlorobionts *Chlamydomonas reinhardtii, Physcomitrella patens *and *Selaginella moellendorffii *were compared to the reference seed plant *Arabidopsis thaliana *and the sequences were evaluated with respect to function and localization. The analysis follows up with previous comparison of gene families [13,17,23], focusing on the composition of the chloroplast antioxidant system.

### Primary data mining for chloroplast APx, Prx and GPx

To minimize the difficulties of sequence homology-based genome annotation, data mining was started with screening *Selaginella moellendorffii*, *Physcomitrella patens *and *Chlamydomonas reinhardtii *EST databases for sequences with similarity to cDNAs encoding *Arabidopsis thaliana *APx, GPx and Prx using BLASTN and TBLASTX [29]. In this step all information was collected irrespective of the localization of the encoded proteins. Subsequently, the collected ESTs were clustered by ClustalW2.0 based on nucleotide sequence similarity.

These first clusters indicated 7, 20 and 11 APx for Chlamydomonas, Physcomitrella and Selaginella, respectively, and 3, 3 and 3 2CP, 1, 3 and 2 PrxQ and 3, 5 and 3 PrxII and 9, 8 and 10 GPx (data not shown). To analyze the primary datasets for gene duplication and alternative splicing all non-perfectly matching EST sequences were removed from the EST assembly. The excluded ESTs were subsequently re-tested for their similarity to other or additional EST clusters.

From each refined cluster, the consensus sequence was predicted from the sequence alignment and was used as a working model for a gene-specific class of transcripts. To test whether the particular consensus encodes a peroxidase, the amino acid sequences were compared with the amino acid sequences of characterized Arabidopsis proteins [13,14,17,22,30,31].

Finally, the genomes of Chlamydomonas, Physcomitrella and Selaginella were screened by sequence similarity based on TBLASTX analysis for so far not identified paralogs and pseudogenes. This analysis resulted in 22 additional putative peroxidase sequences (data not shown). The collected set of sequence data was subsequently evaluated with respect of chloroplast targeting.

### N- and C-terminus prediction

Our study specifically focused on identification of genes encoding chloroplast APx, GPx and Prx. EST-assembly and EST-alignment-based sequence retrieval and prediction resulted in various sequences with atypical start or stop codons indicating that the sequences lack information on the protein C- and N-termini, such as N-terminal organellar targeting signals and C-terminal membrane anchors, or that the ESTs reflect incomplete or wrong splicing or genomic contaminations of the EST libraries.

The nucleotide and amino acid sequence conservation of N-terminal targeting signals and Cterminal membrane anchors are often low between species, because the encoded protein domains are mainly structurally conserved [28]. To overcome this detriment, BLASTN searches in EST databases were performed with the 5'- and 3'- ends of the previously retrieved cluster consensus sequences in the third round of data mining. All newly collected ESTs were tested for their cluster compatibility by BLASTN sequence comparison. From the refined EST-clusters, the hypothetical cluster consensus cDNA was calculated using the ClustalW2.0 multiple sequence alignment tool [32].

For all remaining EST clusters without N-terminal transit peptides, the genomic DNA was screened 2000 bp upstream of the EST covered regions for transcriptional start sites and/or additional exons by DBTSS [33] and FEX [34] (data not shown). In parallel, since identification of N-terminal targeting signals turned out to be the most difficult step, the three reading frames of the 2000 bp upstream genomic sequences were translated into amino acid sequences and screened for sequence criteria indicating organellar targeting sequences [28]. Special attention was given to the ratio of hydroxylated and positively charged amino acid residues.

Similarly, 2000 bp downstream of all predicted APx sequences, the respective genomic DNA was screened for transmembrane helices by PredictProtein [35] and retested by TMHMM [36], TMPro [37] and WHEEL http://cti.itc.virginia.edu/~cmg/Demo/wheel/wheel_instructions.html following three frame translation. For any sequence indicating a putative N-terminal or C-terminal extension the EST databases were screened by BLASTN for so far not identified ESTs.

Finally, any predicted chloroplast peroxidase was scanned for its chloroplast targeting probability using TargetP and ATP. These prediction algorithms base on sequence patterns of chloroplast proteins from higher plants (TargetP; [38]) and Physcomitrella (ATP; [39]). In *Chlamydomonas reinhardtii *a single large chloroplast almost completely surrounds the nucleus. As shown e.g. for LHCII and RbcS, which are post-translationally targeted to chloroplasts by strong N-terminal targeting signals in higher plants [40], protein targeting frequently takes place by localized cytoplasmic mRNA translation and demands for less strong chloroplast targeting signals [41]. Consequently, the cut-off values were evaluated for Chlamydomonas based on sequence characteristics (Table 1). Finally, the deduced and validated sequences tags were aligned with the clustered ESTs to predict the full length cDNAs, which were translated into amino acid sequences and compared with genomic DNA. For the four species, sequence analysis resulted in prediction of 49 ORFs encoding chloroplast Prx, GPx or APx. For 37 ORFs gene-specific ESTs were observed. The 12 remaining ORFs may be silent pseudogenes, only weakly expressed or expressed only at certain times or in response to specific stimuli.

**Table 1:** Complexity and identity of APx and Prx from Arabidopsis thaliana (At), Selaginella moellendorffii (Sm), Physcomitrella patens (Pp) and Chlamydomonas reinhardtii (Cr).

	Enzyme (chloroplast-targeted)	Working name	Length [aa]	ATP/TargetP value	Expression level *A. thaliana*: Array-data Other plants: EST counts	Gene code/Location in genome
***Arabidopsis thaliana***						

	2-Cys-Peroxiredoxin	At2CPA	267	0.49641/0.988	3184.8	At3G11630

	2-Cys-Peroxiredoxin	At2CPB	274	0.71435/0.971	1222.76	At5G06290

	Peroxiredoxin Q	AtPrxQ	217	0.82014/0.904	1835.08	At3G26060

	Peroxiredoxin type II	PrxIIE	235	0.63576/0.936	861.2	At3G52960

	stromal ascorbate peroxidase	AtsAPx	372	0.86202/0.864	328.86	At4G08390

	thylakoid ascorbate peroxidase	AttAPx	426	0.62140/0.983	403.91	At1G77490

	glutathione peroxidase	AtGPx1	237	0.53344/0.970	986.38	AT2G25080

	glutathione peroxidase	AtGPx7	234	0.74449/0.969	66.18	AT4G31870

***Selaginella moellendorffii***						

	2-Cys-Peroxiredoxin	Sm2CPA.1	275	0.53959/0.943	67	96: 53633-52442

	2-Cys-Peroxiredoxin	Sm2CPA.2	275	0.53959/0.945	60	46: 1467726-1468926

	2-Cys-Peroxiredoxin	Sm2CPB.1	323	0.653/0.44061	0	34: 503029-503989

	2-Cys-Peroxiredoxin	Sm2CPB.2	317	0.725/0.44061	0	18: 2038503-2040005

	Peroxiredoxin Q	SmPrxQA.1	221	0.51415/0.930	21	21: 1420112-1421465

	Peroxiredoxin Q	SmPrxQA.2	221	0.54575/0.928	7	31: 310820-309427

	Peroxiredoxin Q	SmPrxQB.1	185	0.383/0.82512	0	51: 178527-179338

	Peroxiredoxin Q	SmPrxQB.2	185	0.410/0.82512	0	55: 755418-756216

	Peroxiredoxin type II	SmPrxIIA.1	241	0.60416/0.975	12	4: 3124723-3124001

	Peroxiredoxin type II	SmPrxIIA.2	241	0.47769/0.954	15	91: 178039-179068

	stromal ascorbate peroxidase	SmsAPx.1	349	0.58519/0.689	3	65: 2501511-248461

	stromal ascorbate peroxidase	SmsAPx.2	349	0.58519/0.690	6	72: 297944-296262

	thylakoid ascorbate peroxidase	SmtAPx.1	401	0.49697/0.916	9	7: 2423527-2425329

	thylakoid ascorbate peroxidase	SmtAPx.2	407	0.58844/0.704	10	36: 1259197-126991

	glutathione peroxidase	SmGPxA.1	253	0.60188/0.523	26	107:189067-190114

	glutathione peroxidase	SmGPxA.2	253	0.60188/0.523	26	40:1392048-1393091

	glutathione peroxidase	SmGPxB.1	208	0.47597/0.012	8	57:785613-784714

	glutathione peroxidase	SmGPxB.2	208	0.67471/0.001	0	0:6625208-6626109

	glutathione peroxidase	SmGPxC.1	169	0.46928/0.111	5	0:5532358-5531571

	glutathione peroxidase	SmGPxC.2	169	0.46928/0.111	1	12:261185-260399

***Physcomitrella patens***						

	2-Cys-Peroxiredoxin	Pp2CPA	283	0.52994/0.968	48	30: 2368256-2370888

	2-Cys-Peroxiredoxin	Pp2CPB	n. a.	0.42121/0.945	n. a.	1st part: scaff. 1139; 2nd part: scaff. 257

	Peroxiredoxin Q	PpPrxQA	220	0.30874/0.809	13	233: 663095-664648

	Peroxiredoxin Q	PpPrxQB	220	0.62288/0.977	7	30: 1873696-1872791

	Peroxiredoxin Q	PpPrxQC	220	0.63957/0.970	13	95: 290862-289423

	Peroxiredoxin type II	PpPrxIIA	266	0.54575/0.898	30	52: 1909032-1908279

						

	Peroxiredoxin type II	PpPrxIIB	249	0.47646/0.688	24	1: 1454912-1454166

	thylakoid ascorbate peroxidase	PptAPx	441	0.49166/0.842	161	424: 212264-213717

						

	glutathione peroxidase	PpGPxA.1	248	0.43328/0.869	10	115:583295-585354

	glutathione peroxidase	PpGPxA.2	258	0.43328/0.869	8	115:583295-585241

	glutathione peroxidase	PpGPxB	289	0.53257/0.889	0	313:171870-174548

***Chlamydomonas reinhardtii***						

	2-Cys-Peroxiredoxin	Cr2CPA	236	0.30495/0.669	JGI:121; ChlamyDB:10	3: 2066498-2065265

	2-Cys-Peroxiredoxin	Cr2CPB	199	0.15565/0.014	JGI:19; ChlamyDB:5	5: 332991-334500

	2-Cys-Peroxiredoxin	Cr2CPC	184	0.13318/0.033	0	105: 86681-89539

	Peroxiredoxin Q	CrPrxQ	197	0.44733/0.407	0	8: 1690093-1692044

	Peroxiredoxin type II	CrPrxIIC	195	0.42189/0.105	JGI:3; ChlamyDB:2	1: 570401-572219

	stromal ascorbate peroxidase	CrsAPxA	327	0.44733/0.360	JGI:5; ChlamyDB:1	7: 1855857-1852628

	stromal ascorbate peroxidase	CrsAPxB	376	0.44733/0.24	0	35: 346143-341772

	glutathione peroxidase	CrGPxA	202	0.50985/0.057	0	7:731625-733049

	glutathione peroxidase	CrGPxB	259	0.65448/0.649	0	95:208056-205010

	glutathione peroxidase	CrGPxC	212	0.42097/0.297	JGI:2; ChlamyDB:0	4:1977798-1976386

### Comparison of predicted sequences with data resources of PeroxiBase

Finally the predicted sequences were compared with PeroxiBase [42], which is a novel database summarizing EST-assembly based predictions for plant peroxidases of various cellular compartments. For 22 of the here described 45 ORFs (Table 1), entries are listed in PeroxiBase (Table 1).

While the coverage of Arabidopsis, Physcomitrella and Chlamydomonas chloroplast peroxidases was good in PeroxiBase, all except two of the chloroplast peroxidases of *Selaginella moellendorffii *were newly predicted in our study (Table 1). In addition, one notlisted (possibly silent) Chlamydomonas ORF was described and two Physcomitrella PrxII were newly predicted (Table 1). Based on our EST-assembly analysis the N-termini of one Physcomitrella chloroplast APx (PeroxiBase-Entry 2497) and of one Selaginella 2CP (PeroxiBase-Entry 6217) were corrected (Table 1). Furthermore, for a Physcomitrella 2CP (PeroxiBase-Entry 6396), which is encoded by an incompletely assembled genome domain in Cosmoss, the C-terminus was extended by combination of sequence information split on two sequencing units. Additionally, so far not covered short sequence traces were inserted in the PpPrxQC (PeroxiBase-entry 6324) and CrsAPxB (PeroxiBase-entry 2286) sequences and the sequence translation was corrected for the Chlamydomonas GPxA ORF by addition of missing G_137 _(Table 1).

From this study, 10 ORFs for chloroplast peroxidases were predicted for the each of the two sequenced haplotypes of *Selaginella moellendorffii*, 11 chloroplast peroxidase ORFs for *Physcomitrella patens *and 10 for *Chlamydomonas reinhardtii *(Table 1). They were compared to six well described Arabidopsis chloroplast peroxidases [5,13,14,19,20,22]. Specific characteristics of the here predicted chloroplast peroxidases are:

### Ascorbate peroxidases

#### Gene copy number and predicted protein localization

Many higher plants encode two types of chloroplast ascorbate peroxidases: One is localized in the stroma (sAPx) and one anchored in the thylakoid membrane by a C-terminal transmembrane helix (tAPx) [11,20]. In our study, the predicted chloroplast APx sequences were sub-classified into membrane anchored and soluble isoforms based on TMHMM [36] and TMPro analysis for transmembrane helices [37]. The lipophilicity of the outer helix surface was tested by WHEEL-analysis.

In *Arabidopsis thaliana *stromal and thylakoid ascorbate peroxidases are encoded by two distinct genes, At4g08390 (sAPx) and At1g77490 (tAPx) [20]. In contrast, e.g. in tobacco, spinach and pumpkin stromal and thylakoid ascorbate peroxidases result from alternative splicing of the same gene [20,43,44]. In rice, the sAPx gene triplicated (OsAPx5 - 7; [45]), while there is only one gene encoding a tAPX (OsAPx8). The comparison demonstrates dynamics in the gene copy number over time.

After removal of the extraplastidic isoforms, EST analysis of two haplotypes of *Selaginella moellendorffii *resulted in four clusters encoding chloroplast APx. Two clusters encode sAPx and two tAPx with C-terminal transmembrane helices. The sequences within each pair differ only in 7 - 9 bp. Comparison of the gene environment revealed that the predicted sequences are surrounded by the same genes. Thus, the predicted similar consensus cDNAs represent homologous genes from the two haplotypes, of which genomic DNA was extracted for sequencing [46]. It is concluded that each haplotype encodes one stromal (SmsAPx) and one thylakoid APx (SmtAPx) (Table 1).

**Figure 1:**
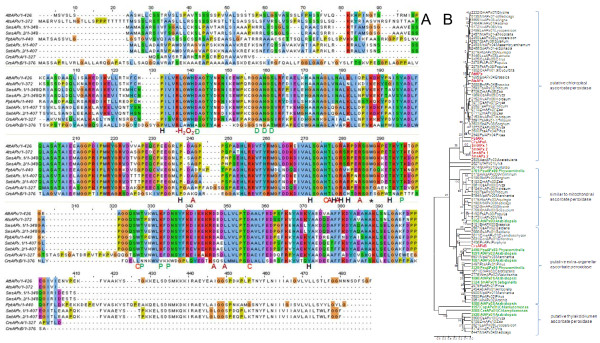
**Comparison of ascorbate peroxidase amino acid sequences**. A: Amino acid sequence alignment of ascorbate peroxidases (APx) from *Arabidopsis thaliana *(At), *Selaginella moellendorffii *(Sm), *Physcomitrella patens *(Pp) and Chlamydomonas *reinhardtii *(Cr). The label "H_2_O_2_" marks the H_2_O_2_-binding site, "C" the amino acids involved in formation of the catalytic site, "P" the proximal and "D" the distal cation binding site and "H" the heme binding amino acids. B: Phylogramme of APx proteins. The proteins depicted in Fig. 1A are marked in red. They are compared to all putative full-length organellar APx listed in PeroxiBase and a selection of extra-organellar APx. The tree was calculated based on the neighborhood joining algorithm. Additional in PeroxiBase predicted, but not in Fig. 1A listed APx from *Arabidopsis thaliana*, *Physcomitrella patens *and *Chlamydomonas reinhardtii *are labeled in green. For all PeroxiBase-data the data base IDs are presented in the labels. The numbers represent bootstrap values for the branches as calculated based on 500 bootstraps. Maximum parsimony and minimum evolution trees are shown in the additional files 1 and 2.

In contrast to the phanerophytes Arabidopsis and Selaginella, the bryophyte *Physcomitrella patens *encodes only one APx (Table 1). The amino acid composition of the C-terminal extension gives strong indications for a 22 amino acid long transmembrane helix (probability = 82.8% according to TMHMM; position aa^417^-aa^440 ^in the 441 amino acid long pre-protein; aa^458 ^- aa^480 ^in fig. 1A), demonstrating that the only ascorbate peroxidase is a tAPx. In the NCBI database the sequence tag is annotated as sAPx (BQ042082) because the automated prediction lacks the sequence information on the transmembrane anchor. Alignment of ESTs gave no indication for alternative splicing (data not shown), demonstrating that in Physcomitrella all chloroplast APx activity is thylakoid-bound and that the moss does not encode a sAPx.

On the contrary, *Chlamydomonas reinhardtii *expresses only a sAPx (CrsAPxA). TMHMM- [36] and AmphipaSeeK-screens [47] for transmembrane helices were negative (data not shown). In the Chlamydomonas genome, a second ORF for a putative soluble APx (CrsAPxB) was observed. However, CrsAPxB is not covered by ESTs.

Comparison of the stromal domain of the mature proteins (aa^134 ^- aa^406 ^in fig. 1A) separates the two Chlamydomonas APx sequences in a species-specific manner (Fig. 1B and additional files 1 and 2). CrsAPxA clusters with chloroplast APx. In contrast, for CrsAPxB only homologues from fungi and non-green algae (non-chlorobionts) were reported in PeroxiBase. According to sequence homology, CrsAPxB is classified as a hybrid ascorbatecytochrome c peroxidase (Entry 2286; Table 1).

In addition to the two Chlamydomonas APx reported here, PeroxiBase lists a third APx with similarities to putative chloroplast APx for Chlamydomonas (ID 2805). We excluded the gene in our EST-assembly analysis, because parts of the catalytic site, e.g. aa^322 ^- aa^380 ^(numbers relative to alignment shown in fig. 1A) are strongly modified in the algal protein indicating that the enzyme has no ascorbate peroxidase function.

#### Exon-intron structure

Evolution primarily selects for the functionality of proteins. Most non-sense mutations get discarded. Intron insertions, deletions and splice site shifts can be tolerated more easily. Consequently, analysis of gene structures can reveal additional information on the phylogenetic relationship between genes of different organisms [47].

**Figure 2:**
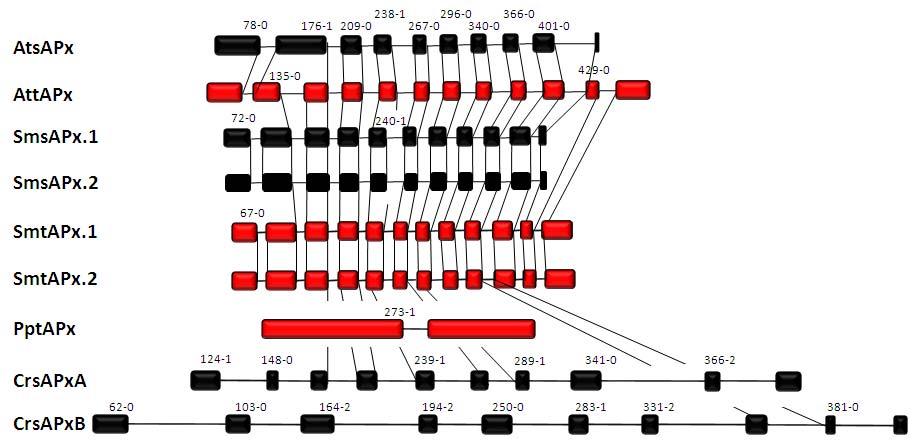
**Relative exon and intron lengths of chloroplast APx in *Arabidopsis thaliana *(At), Selaginella moellendorffii (Sm), *Physcomitrella patens *(Pp) and *Chlamydomonas reinhardtii *(Cr)**. tAPx are shown in red, sAPX in black. The vertical lines connect corresponding splice sites. The numbers represent positions of corresponding amino acids in the alignment shown in fig. 1A and the relative splice sites within the corresponding codon.

In *Arabidopsis thaliana*, the coding sequence of sAPX is split into 10 exons and the tAPX is encoded by 12 exons (Fig. 2. Nine splice sites are conserved demonstrating that the two APx genes are of common origin. The stop-codon of sAPx is replaced by a glutamate codon in AttAPx and the open reading frame extents into an additional exon (exon 12; Fig. 2). This exon encodes the 22 amino acids long C-terminal transmembrane helix. In AttAPx an additional intron is inserted in the 0-position of the codon for aa^135 ^(numbers according to the position in fig. 1A). This intron is missing in AtsAPx (Fig. 2).

The exon-intron-structure of Selaginella tAPx resembles that of Arabidopsis tAPx in both haplotypes. The splice sites are widely conserved to Arabidopsis tAPx from the second site onwards (Fig. 2). Like in AttAPx, the sequence encoding aa^82 ^- aa^209 ^(Fig. 1A) is split into two exons (Fig. 2). The similar gene structure indicates that SmtAPx, SmsAPx and AttAPx evolved very likely from a common ancestor gene. As species-specific variation, the introns are all shorter in Selaginella than in Arabidopsis.

Compared to AtsAPx, SmsAPx has an extended acidic and hydroxylated C-terminus (DESTS; aa^406^-aa^410 ^in Fig. 1A). Its sequence shows no homology to the C-terminus of any tAPx or sAPx listed in PeroxiBase (data not shown) or in enzymes identified in our analysis (Fig. 1A) and, consequently, is a specific extension of SmsAPx.

In the green alga Chlamydomonas the two sAPx genes have 10 exons each (Fig. 2). The introns were much longer than in AtsAPx. In total, the difference in intron length increases the size of the genes by a factor of 1.5 - 2. In CrsAPxA, from the third exon downstream, four splice sites are conserved with *Arabidopsis thaliana *APx and two differ only in one codon length (Fig. 2). For CrsAPxB, the gene structure analysis revealed only one conserved splice site (aa^366-0^) (Fig. 2) confirming the less related nature of the gene.

PptAPx, which is the only chloroplast APx of *Physcomitrella patens*, has the most atypical gene structure within the group of analyzed genes. The PptAPx hnRNA has only one splice site. It is located approximately in the mid of the transcript at the non-conserved position 273-1 (relative to the amino acid position depicted in Fig. 1A) (Fig. 2). The GC-content of the PptAPx transcript is with 52.0% increased if compared to AttAPx transcript (46.3%) and to the average GC-content of Physcomitrella genes (31.7 - 47.7%; [48]). The PptAPx gene is flanked by large footprints of Angela LTR retrotransposons http://www.cosmoss.org, indicating that the only APx gene of Physcomitrella encoding a plastid isoform is of retrotransposonal origin and not directly related to the described APx genes from the other analyzed species.

#### Expression analysis

**Figure 3:**
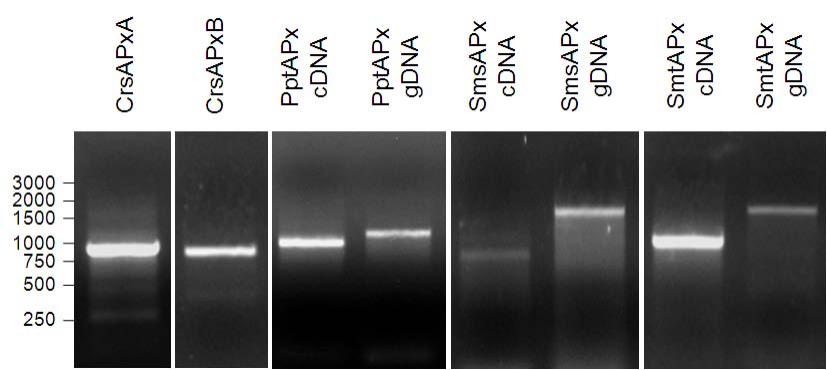
**PCR amplification of full-length genomic DNA (gDNA) and cDNA fragments encoding the predicted stromal and thylakoid-bound ascorbate peroxidases in *Chlamydomonas reinhardtii*, Physomitrella patens and *Selaginella moellendorffii *proving the predicted cDNA lengths**.

To test the predicted APx genes for expression and the predicted transcript length, RNA was isolated from *Chlamydomonas reinhardtii*, *Selaginella moellendorffii *and *Physcomitrella patens *and transcribed into cDNA. Subsequently, the cDNAs were tested for sAPx or tAPx expression by saturating PCR (40 cycles at optimal temperature) with primers binding to the gene specific sequences encoding the N-terminus and the C-terminus of the mature protein. With all samples single products with predicted lengths of 888 bp for CrsAPxA, 900 bp for CrsAPxB, 1073 bp for PptAPx, 825 bp for SmsAPx and 1011 bp for SmtAPx were amplified (Fig. 3), confirming the gene predictions (Fig. 2) and demonstrating expression of all predicted genes. Since RNA isolation is different for the various species and results in different yields, quantitative comparison of transcript abundances was omitted.

#### Characteristics of the predicted proteins

Among the identified chloroplast APx, the amino acid residues forming the catalysis triad (H^276^, D^356 ^and W^327^) are conserved in all identified proteins (Fig. 1A; except CrsAPxB). All proteins have also R^137^, W^140 ^and H^141 ^in common (Fig. 1A), which coordinate the H_2_O_2 _molecule in the active site [49]. In response to excess H_2_O_2_, W^140 ^can crosslink with the heme, which irreversibly inhibits the enzyme [50]. Regarding the heme binding site, in Physcomitrella tAPx and Chamydomonas sAPx position R^282 ^is replaced by a H, like it is in many cytosolic APx [49]. All other amino acids important for heme binding (P^133^, P^238^, L^272^, H^276^, L^278^, G^279^, R^280^, S^286^, and Y^372^) [4,51] (labeled "H" in Fig. 1A) are conserved within the examined species.

The amino acids involved in ascorbate binding (T^277^, A^241^, G^279^, R^285^, D^347^, and L^350^) (labeled "A" in Fig. 1A) [4,49,51] are widely conserved like several unspecified residues (e.g. L^138^, G^144^, T^145^, Y^146^, K^148^, I^150^, E^152^, W^153^, P^154^). In total 63 - 79% amino acid identity for sAPx and 69 - 79% identity for tAPx reflect a high overall similarity between Arabidopsis, Selaginella and Physcomitrella chloroplast APx. Many amino acid substitutions conserve the chemical properties of the protein (Fig. 1A), for example E^341 ^is replaced by the also acidic amino acid D in both SmsAPx and AttAPx and S^195 ^is substituted by T and L^351 ^by V in PptAPx. The most prominent amino acid exchange is the replacement of W^288 ^by F in CrsAPxA, CrsAPxB und PptAPx since the F^288^W substitution has been proposed to provide higher ascorbate specificity to chloroplast APx compared to cytosolic APx [20].

**Figure 4:**
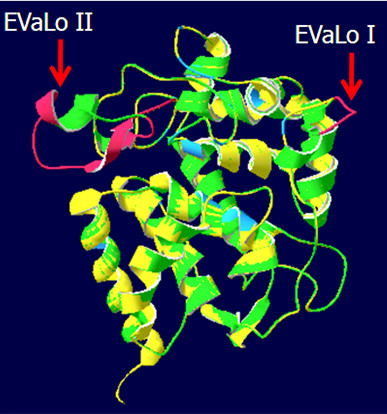
**Superimposition of AtsAPx (yellow) and CrsAPxA (green) structures**. The two loops formed by the insertions aa^244^-aa^248 ^(EVaLo I) and aa^303^-aa^321 ^(EVaLO II) are shown in red.

Ascorbate peroxidases bind one or two cations on the protein surface, which are involved in heme coordination [49]. The distal cation binding site (D^142^, G^158^, N^160^, S^162^) ("D" in Fig. 1A) is conserved in all APx encoded by expressed genes. In the proximal cation binding pocket (T^296^, T^328^, K^333 ^and D^335^) (labeled "P" in Fig. 1A), K^333 ^is exchanged by E in CrsAPxA. The higher number of negative charges provides a stronger ionic interface for the potassium ion. Superimposition of the modeled structure of CrsAPxA with AtsAPx (Fig. 4) shows that two short insertions (aa^244^-aa^248^, aa^303^-aa^321 ^in fig. 1) form loops, designated "Evolutionary VAriable LOops I and II" [EVaLo I (aa^244^-aa^248^) and EVaLo II (aa^303^-aa^321^)], on the protein surface (Fig. 4).

### 2-Cys peroxiredoxins

#### Gene copy number

Like *Arabidopsis thaliana *(At2CPA: At3g11630; At2CPB: At5g06290), *Physcomitrella patens *encodes two 2CP (per haploid genome) (Table 1). N-terminal targeting signals indicate that both are chloroplast-targeted isoforms. Due to a gap between the genome scaffolds 1139 and 257 http://www.cosmoss.org, no gene model was predictable for Pp2CPB.

**Figure 5:**
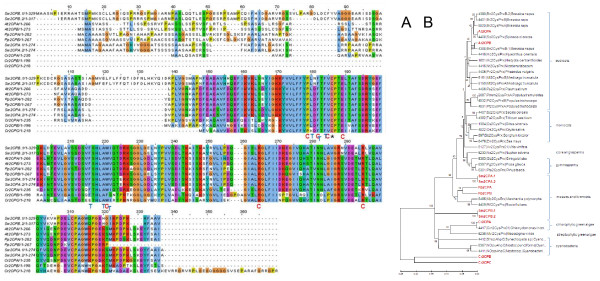
**Comparison of 2-Cys peroxiredoxin amino acid sequences**. A: Amino acid sequence alignment of 2-Cys peroxiredoxins (2CP) from *Arabidopsis thaliana *(At), *Selaginella moellendorffii *(Sm), *Physcomitrella patens *(Pp) and Chlamydomonas *reinhardtii *(Cr). The peroxidatic and resolving cysteine residues are labeled with "*", "T" indicates the amino acid residues involved in decamer formation and "C" the residues forming the catalytic site. B: Phylogramme of the 2CP sequences shown in Fig. 5A (red) and additional 2CP from chlorobionts and cyanobacteria as listed in PeroxiBase [96]. The tree was calculated based on the neighborhood joining algorithm. For all PeroxiBase-data the data base IDs are presented in the labels. The numbers represent bootstrap values. Maximum parsimony and minimum evolution trees are shown in the additional files 3 and 4.

The two haplotypes of Selaginella encode two pairs of almost identical 2CP, designated Sm2CPA.1/2 and Sm2CPB.1/2. For the Sm2CPB genes no ESTs were observed (Table 1), indicating that they are putatively not, only weakly or only under defined circumstances expressed. According to gene models presently provided by the Selaginella databases, the Sm2CPB start-codons were predicted corresponding to position 118 or 145 (relative to the positions in fig. 5A) and lacking the N-terminal extension with chloroplast targeting propensity. A homologous, but incomplete putative N-terminal extension was observed for Sm2CPB.2 (Fig. 5A).

**Figure 6:**
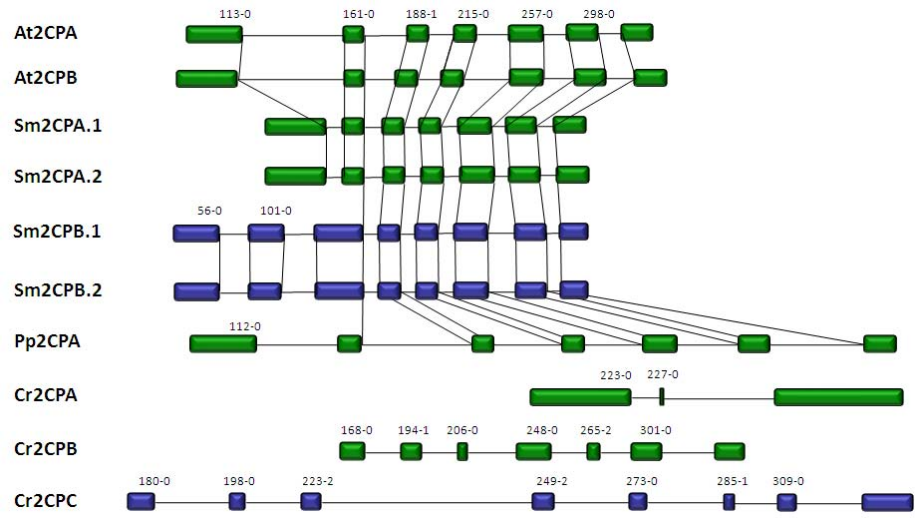
**Proportional comparison of the gene structures of 2CP in *Arabidopsis thaliana *(At), Selaginella moellendorffii (Sm), *Physcomitrella patens *(Pp) and *Chlamydomonas reinhardtii *(Cr)**. ESTcovered 2CP genes are shown in green, putatively non-expressed in blue. The vertical lines connect corresponding splice sites. The numbers represent positions of corresponding amino acids in the alignment shown in Fig. 5A and the relative splice sites within the corresponding codon.

In the genome of *Chlamydomonas reinhardtii *three open reading frames for 2CP were identified (Table 1). Cr2CPA is identical to PRX1 and Cr2CPB to PRX2, which were previously described by Dayer et al. [16]. For the Cr2CPA gene 121 ESTs were observed in the JGI database and 10 in ChlamyDB, indicating that the gene is strongly expressed relative to other peroxidases in Chlamydomonas. The deduced protein sequence shows a typical N-terminal chloroplast targeting signal. For Cr2CPB, which was previously predicted to be cytosolic (or flagellar) [16], only 19 ESTs were observed in the JGI database and 5 in ChlamyDB (Table 1), suggesting that the gene is less expressed. The N-terminus of the protein is exceptionally short if compared to 2CP from other plants (Fig. 5A). Chlamydomonas genes often have longer introns than those of Arabidopsis and Selaginella (see e.g. Fig. 2 and 6). Thus, for the Cr2CPB gene 4000 bp upstream sequence were screened for a putative additional exon, but no indications were found for a putative targeting signal with TargetP-values higher than 0.3 (data not shown). It indicates that the Cr2CPB gene, although only weakly expressed, may encode the first identified plant non-chloroplast 2CP consistent with the protein localization suggested by Dayer et al. [16]. However, as shown for RbcS and LHCII, protein import into chloroplasts is strongly regulated by localized translation in Chlamydomonas [41], which does not necessarily need strong chloroplast import signals.

Furthermore, our study identified a third putative, but not-EST-covered 2CP gene, Cr2CPC, on scaffold 105 (Table 1), which shares higher similarity with Cr2CPB than with Cr2CPA (Fig. 5A and 5B). According to the neighborhood joining (Fig. 5B) and minimal evolution algorithms (Additional file 3), Cr2CPA clusters into the same branch as cyanobacterial 2CP, while Cr2CPB and Cr2CPC form an independent outgroup. Only the maximum parsimony algorithm (Additional file 4), which accounts for the most parsimonious explanation based on weighting each amino acid position discretely [52], clusters the three *Chlamydomonas reinhardtii *2CP with a 2CP known from *Chlamydomonas incerta *in a group which is closely related to cyanobacterial 2CP.

#### Exon-intron structure

Like for APx, the gene structures of the 2CP were analyzed by comparison of cDNA sequences with genomic DNA (Fig. 6). The two A. *thaliana *2CP have very similar structures with conserved exon-intron borders (Fig. 6). The first exon of At2CPB, which encodes the less conserved chloroplast targeting signal, is eight amino acids longer than in At2CPA. The main difference between the two genes is the length of the introns, which are slightly longer in At2CPB.

A very similar gene structure with seven exons was observed for Sm2CPA (Fig. 6) demonstrating gene conservation between the lycophyte and the seed plant. The introns are all shorter in Sm2CPA than in Arabidopsis 2CP genes, which decreases the total length of the gene by 33%. The splice sites are conserved with those in *A. thaliana *2CP (Fig. 6). The not-EST-covered Selaginella 2CPB gene has eight instead of seven exons due to an additional intron in the corresponding first exon of Sm2CPA (Fig. 6). The splice site between exon2 and exon3 is gene-specific and elongates exon3 by 36 nucleotides. In general, the introns of Sm2CPB are longer and their length is more variable than in Sm2CPA (Fig. 6).

In *Physcomitrella patens *Pp2CPA has seven exons, which are separated by comparably long introns (Fig. 6). From the second splice site (aa^161-0^) onwards, the exon-intron borders are conserved with the two Arabidopsis 2CP. The first border (aa^112-0^) differs by only one amino acid from the border found in the Arabidopsis 2CP (aa^113-0^).

For *Chlamydomonas reinhardtii*, three 2CP genes were detected in the genome. Their gene structure is non-conserved and atypical if compared to Physcomitrella, Selaginella and Arabidopsis 2CP (Fig. 6). Cr2CPA has only three exons while the weakly or not expressed Cr2CPB and Cr2CPC genes have seven and eight exons, respectively, with non-conserved splice sites.

#### Characteristics of the predicted proteins

Despite the variability in gene structures (Fig. 6), the amino acid sequences of the mature 2CP share many conserved positions (Fig. 5A). The catalytic sites around the peroxidatic C^186 ^(Fig. 5A; aa^171 ^- aa^196^), around the resolving cysteine residue C^311 ^(aa ^310 ^- aa^314^) ("*" in fig. 5A) and the active pocket (P^179^, F^182^, V^185^, E^189^, W^221^, R^265 ^and R^295^) ("C" in fig. 5A) [53] are identical in all 2CP for which EST data were observed. Furthermore, the GGLG-motif (aa^229^-aa^232^) is conserved in all species. According to Jönsson et al. [54], the motif is specific for eukaryotic 2CP and stabilizes the folded structure together with the YF-motif (aa^332^-aa^333^), decelerates disulfide formation and increases the sensitivity to H_2_O_2_.

Strongest differences were observed for Cr2CPB, Cr2CPC and Sm2CPB. For example, in the hydrophobic pocket around the resolving C^311^, G^314 ^and K^316 ^are replaced by N and W^221 ^is exchanged by a less bulky F-residue in Cr2CPB. In Brassica 2CP W^221 ^is replaced by G [55] indicating that this position is less important for 2CP function than other amino acids. The dimer interface described by Schroder et al. [53] (R-Q-I-X-V-N-D) is replaced by a Q-HA/S-T-I/V-N-N consensus in most plant 2CP (aa^277^-aa^283^; Fig. 5A) [55], including all ESTconfirmed 2CP identified in this study (Fig. 5).

The interface (L^180^, F^182^, F^184^, F^216^, A^220 ^and W^221^) (labeled "T" in fig. 5A) involved in decamer formation [53] is conserved (Fig. 5A). However, there are a few protein modifications in the dimer and decamer interfaces between the identified proteins: As mentioned before, W^221^, which is also part of the active site, is replaced by F in Cr2CPB. Sm2CPA and Pp2CPA show G^251^N and an I^281^V substitutions and Sm2CPB, which is encoded by a presumably nonexpressed or only weakly expressed gene, has a G^251^R substitution. These two positions are known to be involved in dimer stability [53]. In Sm2CPB also the YF-motif (aa^332^-aa^333^), which is involved in stabilization of the peroxidatic C [54], is replaced by HF and aa^328^-aa^331 ^are missing, suggesting that the putatively less expressed gene does not encode a (fully) functional protein. Sm2CPA and Pp2CPA show charge conservative amino acid substitutions with weak sterical effects (T^243^S: Sm2CPA; D^256^E: Pp2CPA) in the decamer contact phase. At aa^279 ^in the 2CP alignment only Arabidopsis shows a S while Sm2CPA, Sm2CPB, the Physcomitrella 2CP, and Cr2CPA reveal an A residue and Cr2CPB and Cr2CPC an I and V, respectively.

**Figure 7:**
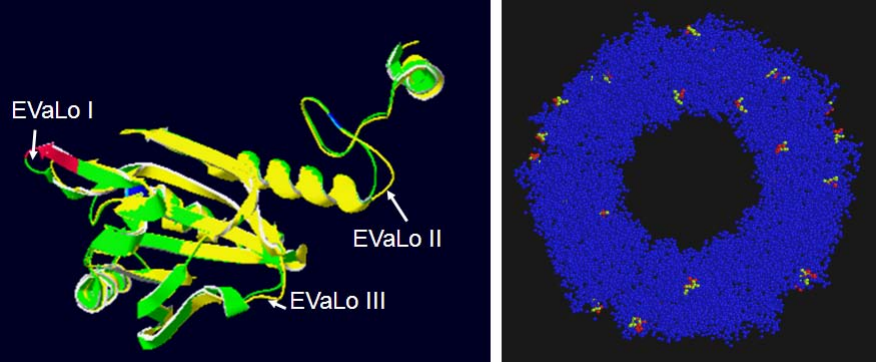
**Superimposition of At2CPA (yellow) and Cr2CPB (green) monomers**. The three amino acid insertion (aa^258 ^- aa^260^) extends the length of a β-sheet and modifies the protein surface. In the decameric toroid structure (right) the three EVaLo-s modify the inner and outer ring surface (red-green labels in the right figure).

Cr2CPB shows a specific three amino acid long insertion at positions aa^258 ^- aa^260 ^(Fig. 5A), which extends a β-sheet on the protein surface (EVaLo I in Fig. 7). In this protein, like in Cr2CPC and partially also in Cr2CPA, charged amino acids are atypically substituted: negatively charged D^148^, E^150 ^and Q^298 ^are substituted by positively and uncharged residues and E^201 ^by R, K or A. Uncharged S^228 ^and L^328 ^are substituted by K, E, R and G, and K^161 ^(by E), K^163 ^(by T/S), K^202 ^(by D, A), K^227 ^(by T/N), K^272 ^(by P), K316 (by N) and K327 (by T/E), which are otherwise widely conserved throughout the 2CP family [55,56], and K^170 ^is deleted (Fig. 5A).

The C-terminus, which is involved in the attachment of 2CP to membranes [18,53], is more hydroxylated in Cr2CPB due to S/T-substitutions at position 334, 335 and 337 (Fig. 5A). In Cr2CPC, which is not covered by ESTs, the C-terminus is extended by a G- and P-rich 33 amino acid long peptide. Secondary structure analysis gave no indications for strong structural features, such as α-helices or β-sheets (data not shown) indicating a long unstructured C-terminal tail, such as in animal and yeast 2CP [53,57].

Consistent with the degree of primary structure conservation, modeling of the 3D-structures indicate strong differences between Cr2CPB and At2CPA (Fig. 7), while the structure of the other EST-covered 2CP is conserved (data not shown): In Cr2CPB (Table 1) a three amino acid insertion (aa^258 ^- aa^260^) extends and slightly tilts the β-sheet which is involved in formation of the active site (Fig. 7; EVaLo I). Other sequence variations impact on the dynamic loops, designated "Evolutionary Variable Loop" II and III. EVaLo II is located on the outer surface of the 2CP toroid-decamer structure [58]. In contrast, EVaLo III shapes the inner surface of the 2CP-toroid. These predicted modifications indicate a specific surface of Cr2CPB oligomers (Fig. 7 right).

### The atypical 2-Cys peroxiredoxins PrxQ and PrxII

#### Gene copy number

**Figure 8:**
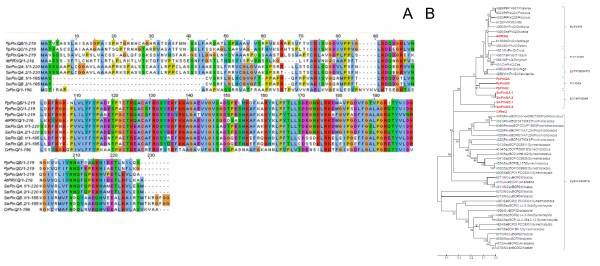
**Comparison of peroxiredoxin Q amino acid sequences**. A: Amino acid sequence alignment of the here analyzed PrxQ from *Arabidopsis thaliana *(At), *Selaginella moellendorffii *(Sm), *Physcomitrella patens *(Pp) and *Chlamydomonas reinhardtii *(Cr). B: Phylogramme of the PrxQ sequences shown in Fig. 8A (red) and putative full-length PrxQ sequences of chlorobiont and cyanobacterial origin as listed in PeroxiBase [96]. The tree was calculated based on the neighborhood joining algorithm. The numbers represent bootstrap values. Maximum parsimony and minimum evolution trees are shown in the additional files 5 and 6.

**Figure 9:**
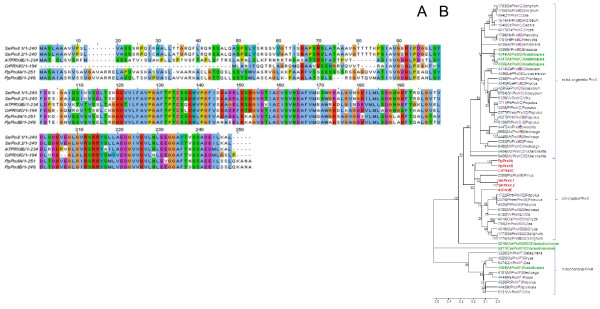
**Comparison of chloroplast type-II peroxiredoxin amino acid sequences**. A: Amino acid sequence alignment of PrxII from *Arabidopsis thaliana *(At), Selaginella moellendorffii (Sm), *Physcomitrella patens *(Pp) and *Chlamydomonas reinhardtii *(Cr). B: Phylogramme of the PrxII sequences shown in Fig. 11A (red) and a selection of PrxII full length sequences listed in PeroxiBase [96]. A *Chlamydomonas reinhardtii *of uncertain location, which is listed in PeroxiBase, but not in our analysis is labeled in green. For all PeroxiBasedata the data base IDs are presented in the labels. The tree was calculated based on the neighborhood joining algorithm. The numbers represent bootstrap values. Maximum parsimony and minimum evolution trees are shown in the additional files 7 and 8.

The atypical 2CP family is comprised of two enzyme groups: PrxQ and PrxII. In *Arabidopsis thaliana*, one out of six PrxII, PrxIIE (At3g52960), is targeted to the chloroplast stroma [14]. The only PrxQ is post-translationally targeted to the thylakoid lumen [19]. According to neighborhood joining (Fig. 8B and 9B), minimum evolution and maximum parsimony trees (Additional files 5, 6, 7 and 8) all identified streptophyte PrxII and PrxQ cluster with chloroplast homologs from other plants.

Like *Arabidopsis thaliana*, *Selaginella moellendorffii *encodes two PrxII proteins with Nterminal targeting signals per haploid genome. One is homologous to chloroplast AtPrxIIE, the other to mitochondrial AtPrxIIF (Fig. 9B). The chloroplast SmPrxII is most similar to the Pinus PrxIIE (Fig. 9B and additional files 7 and 8).

In contrast, two ORFs encoding PrxQ were identified (per haploid genome) in the genome of Selaginella indicating a gene duplication. For SmPrxQA 7 ESTs were counted indicating that the gene is functionally active. No EST was observed for SmPrxQB, which is less similar to higher plant PrxQ than SmPrxQA (Fig. 8B and additional files 5 and 6).

The bryophyte Physcomitrella expresses three chloroplast PrxQ and two PrxII. The gene number demonstrates an amplification of genes encoding atypical Prx if compared to Chlamydomonas, Selaginella and Arabidopsis (Fig. 9; Table 1). According to the calculated phylogenetic trees (Fig. 8B) and in contrast to other Prx genes the three PrxQ proteins, especially PpPrxQA, show species-specific sequence characteristics (Fig. 8B).

Consistent with Dayer et al. [16], one chloroplast PrxII and one chloroplast PrxQ were observed [PRX5 (here: CrPrxIIC) and PRX6 (here: CrPrxQ)] in *Chlamydomonas reinhardtii *(Table 1; Fig. 8). However, unlike Dayer et al. [16], no EST was detected for PrxQ by using the JGI and ChlamyDB data resources.

#### Exon-intron structure of PrxQ

**Figure 10:**
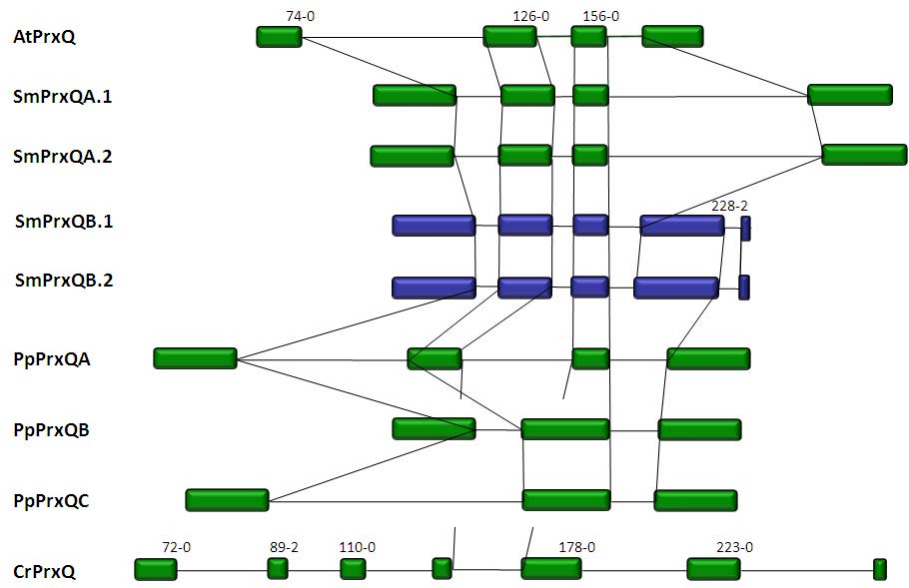
**Gene structures of PrxQ in *Arabidopsis thaliana *(At), *Selaginella moellendorffii *(Sm), *Physcomitrella patens *(Pp) and *Chlamydomonas reinhardtii *(Cr)**. Expressed PrxQ genes are shown in green, non-expressed in blue. The vertical lines connect corresponding splice sites. The numbers represent positions of corresponding amino acids in the alignment shown in Fig. 8A the relative splice site within the corresponding codon.

In Arabidopsis, Selaginella and Physcomitrella two splice sites are conserved within the PrxQ genes (aa^74-0 ^and aa^156-0^) (Fig. 10). PpPrxQA, the Selaginella and Arabidopsis homologs share an additional splice site at amino acid position 126-0 (Fig. 10). CrPrxQ shows a distinct splice pattern. Only one splice site (corresponding to aa^126-0^) is conserved with PpPrxQA, the Selaginella and Arabidopsis homologs and no splice site is conserved with PpPrxQB and PpPrxQC (Fig. 10).

If compared to C. *reinhardtii*, the gene structure of PpPrxQA was more similar to AtPrxQ and SmPrxQ (Fig. 8). It has four exons. The first and the fourth exon have approximately the same length as in the Selaginella homolog. The exons are separated by three introns which are longer than in AtPrxQ, SmPrxQA and SmPrxQB and elongate the entire gene. PpPrxQB and PpPrxQC both have only three exons. Their second exon combines exon2 and exon3 of AtPrxQ, SmPrxQ, and PpPrxQA. In SmPrxQB there is an additional splice site in the last exon (corresponding to aa^228-2^).

#### Exon-intron structure of PrxII

**Figure 11:**
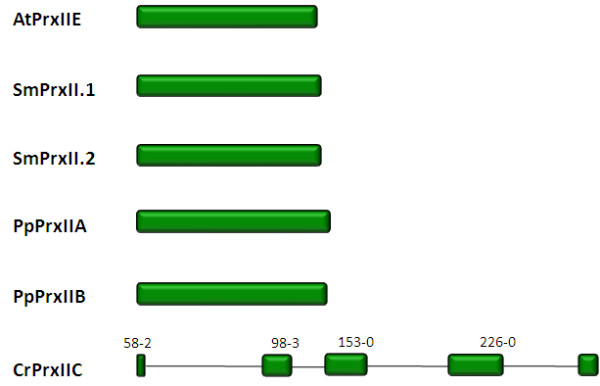
**PrxII gene structures of *Arabidopsis thaliana *(At), *Selaginella moellendorffii *(Sm), *Physcomitrella patens *(Pp) and *Chlamydomonas reinhardtii *(Cr)**. Expressed PrxII genes are shown in green. The vertical lines connect corresponding splice sites. The numbers represent position of corresponding amino acids in the alignment shown in Fig. 11 A and the relative splice sites within the corresponding codon.

The PrxII proteins of *A. thaliana, S. moellendorffii *and *P. patens *are encoded within a single exon (Fig. 11). In contrast, the C. *reinhardtii *PrxII gene has five exons separated by four introns of different sizes.

#### Characteristics of the predicted PrxQ and PrxII proteins

To compare PrxQ and PrxII from Arabidopsis, Selaginella, Physcomitrella and Chlamydomonas, the cDNA sequences were translated. The derived amino acid sequences were aligned by ClustalW2.0 (Fig. 8A and 9A). Atypical plant Prx have so far not been investigated to such detail as APx or typical 2CP. Only few amino acids have been reported to be essential for their activity: The peroxidatic and resolving C residues are characteristic for most Prx. In PrxQ, the conserved C residues are located at positions 123 and 128 (Fig. 8A) and in PrxII at 135 and 160 (Fig. 9A). Additional specific criteria are:

#### PrxQ

Compared to non-plant PrxQ [59], the amino acid sequences are more similar between plant species. In PpPrxQ, SmPrxQ and AtPrxQ many positively charged amino acid residues are conserved (Fig. 8A). K, R, Q and N are protonated under acidic conditions, such as there are in the thylakoid lumen during light periods, where PrxQ is post-translationally targeted to [19]. CrPrxQ lacks negative charges at position 118, 119, 135 (numbers relative to the alignment depicted in Fig. 8A) and positive charges at position 138, 139, 156, 175 and 200. In phylograms, the algal protein is not grouped with PrxQ from higher plants (Fig. 8B and additional files 5 and 6), but defines a distinct group, demonstrating that *Chlamydomonas reinhardtii *expresses a specific type of PrxQ.

In the analyzed plant PrxQ, among the amino acids located close to the active site, aa^120^, aa^122 ^and aa^126 ^are not conserved (Fig. 8). AtPrxQ, PpPrxQA, PpPrxQB, SmPrxQB and CrPrxQ show a T residue at position 120 while it is replaced by the also hydroxylated amino acid S in SmPrxQA and PpPrxQC. More important in respect of enzyme activity might be the exchange of the positively charged Q^126 ^to a negatively charged E in SmPrxQ and CrPrxQ and the G^122^T exchange in PpPrxQB, since they affect the charge distribution close to the active site (C^123 ^- C^128^) (Fig. 8A).

Besides the variation of a hydroxylated amino acid residue in the -3 position (S/T^120^) and P residues in the -2 and -7 position relative to the peroxidatic cysteine residue (C^123^) and R^193^, which has been proposed to be important for dimer stabilization in peroxiredoxins [57], there is too little information on functionally important motifs and amino acids to draw conclusions on enzyme conservation.

**Figure 12:**
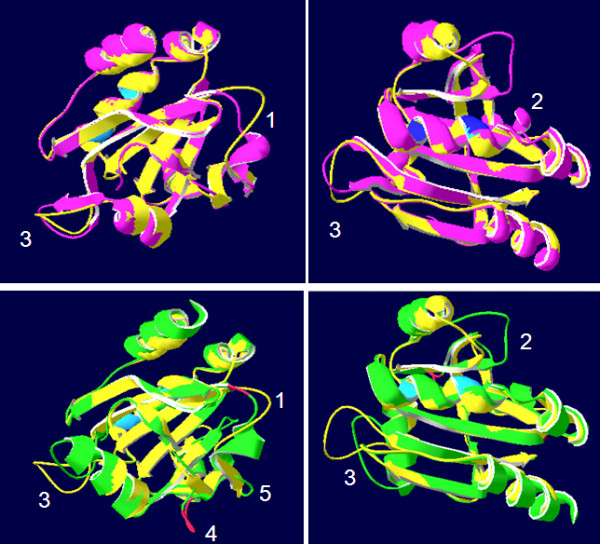
**Superimposition of AtPrxQ (yellow) and SmPrxQA**. 1 (pink) and AtPrxQ (yellow) and CrPrxQ (green) in two views. The positions of the three and five flexible elements on the protein surface are numbered in white for better comparison. The peroxidatic and resolving C are labeled in blue.

The peroxiredoxin fold was investigated by predicting the 3D-structure with SWISS-MODEL (Fig. 12). The parallel β-sheet core and the conserved α-helices give the streptophyte PrxQ proteins a rigid common structure. On the protein surface there are three flexible loops in streptophyte PrxQ (Fig. 12 top) and five loops in the Chlamydomonas PrxQ. The K, R, Q, and N residues, which may be important for the pH-sensitivity of the enzyme, are evenly distributed on the protein surface (data not shown).

#### PrxII

Recent crystal structure analysis of poplar PrxII demonstrated that F^131^, T^132^, P^133^, F^167^, V^168^, A^171^, L^209 ^and R^212 ^form the active site and the interface in PrxII dimers [60]. Apart from position 171, which is replaced by S in SmPrxII of both haplotypes, all positions are conserved in the analyzed species (Fig. 9A).

**Figure 13:**
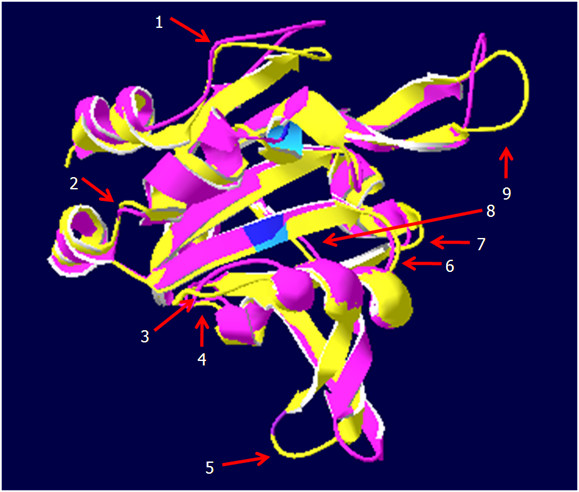
**Superimposition of AtPrxIIE (yellow) and SmPrxII**.1 (pink) showing the peroxidatic and resolving C residues in blue and nine flexible loops (labeled with white numbers).

PrxII interact electrostatically with their electron donors, such as glutaredoxins [61]. From the negative charges suggested to define the electrostatic surface of poplar PrxII [60], the positions 242 (E/D), 243 (E/D) and 231 (E) are conserved. In addition F^131^, which is part of the hydrophobic surface domain, can be found in PrxII of all analyzed species. In contrast, the poplar-specific E^145 ^is replaced by D, S and A. E^178 ^is substituted by small uncharged amino acids V, I and A and L^137 ^partially by S and Q in the here analyzed species (Fig. 9A). 3 D structural comparison showed various flexible loops on the protein surface (Fig. 13), demonstrating that the structure of PrxII proteins is less rigid than the one of the 2CP. Chlamydomonas PrxII showed the shortest N-terminal extension and a deletion of V^206 ^and E^207^.

The two Physcomitrella PrxII have atypical C-termini (Fig. 9A). The eight, instead of three, amino acid long C-terminus has two additional hydroxylated amino acids (S^246 ^and S^247^) and three (Q^249^, K^250 ^and N^252^), instead of one, positively charged residues (Fig. 9A). According to Echalier et al. [62], the C-terminus is exposed on the surface of PrxII proteins. Thus, the atypical tail of PpPrxII may increase the hydrophilicity of the protein. Superimposition of the 3 D structures demonstrates that in *S. moellendorffii, P. patens *and *C. reinhardtii *the position of the peroxidatic C (C^135^) is slightly shifted if compared to AtPrxIIE due to replacement of the positively charged Q^137 ^by S or L (Fig. 9A and 13).

### Glutathione peroxidases

**Figure 14:**
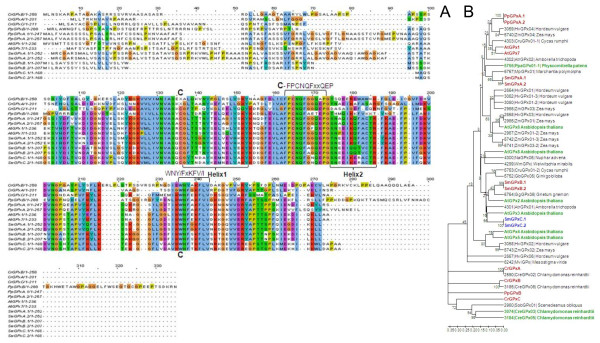
**Comparison of glutathione peroxidase amino acid sequences**. A: Amino acid sequence alignment of GPx from *Arabidopsis thaliana *(At), Selaginella moellendorffii (Sm), *Physcomitrella patens *(Pp) and *Chlamydomonas reinhardtii *(Cr). B: Phylogramme of the GPx sequences shown in Fig. 14A (red) and a selection of plant GPx full length sequences listed in PeroxiBase [96]. For all PeroxiBase-data the data base IDs are presented in the labels. The tree was calculated based on the neighborhood joining algorithm. The numbers represent bootstrap values. Maximum parsimony and minimum evolution trees are shown in the additional files 9 and 10.

Like peroxiredoxins, glutathione peroxidases are broad spectrum peroxidases, which detoxify H_2_O_2 _and a wide range of alkylhydroperoxides [12,63,64]. Plant GPx cluster into the phylogentically ancient group of phospholipid hydroperoxides glutathione peroxidases (PHGPx) [17]. Based on sequence similarities and biochemical characterization they are alternatively designated as a subclass of peroxiredoxins [5]. All of the here described GPx have fully conserved FPCNQF (F^163 ^- F^168^) and WNY/FxKFV/I (W^236 ^- V/I^243^) motifs (Fig. 14A), known for GPx from various plant kingdoms [65].

### Arabidopsis chloroplast GPx

**Figure 15:**
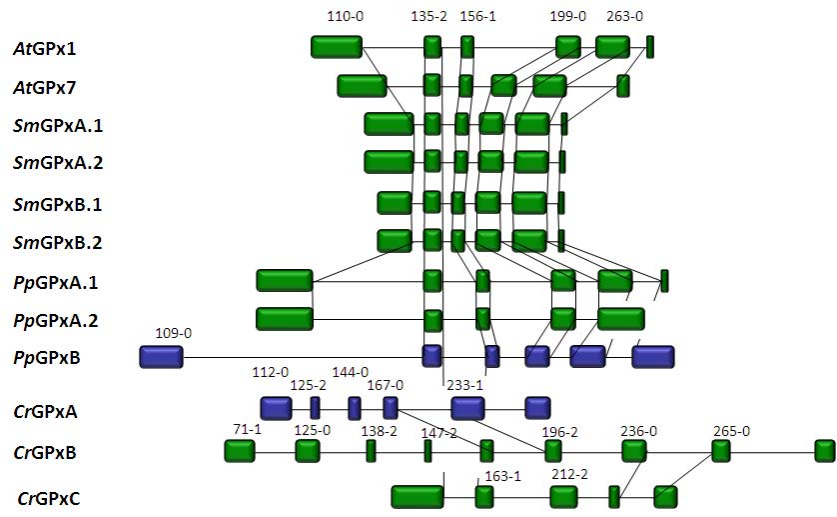
**GPx gene structures of *Arabidopsis thaliana *(At), *Selaginella moellendorffii *(Sm), *Physcomitrella patens *(Pp) and *Chlamydomonas reinhardtii *(Cr)**. Expressed GPx genes are shown in green, putatively non-expressed in blue. The vertical lines connect corresponding splice sites. The numbers represent position of corresponding amino acids in the alignment shown in Fig. 14A and the relative splice sites within the corresponding codon.

**Figure 16:**
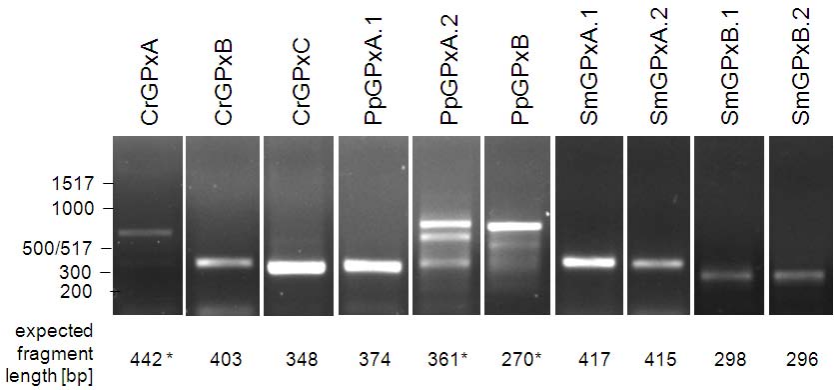
**PCR amplification of cDNA fragments encoding the predicted glutathione peroxidases in *Chlamydomonas reinhardtii*, Physomitrella patens and *Selaginella moellendorffii***. Samples with unspecific bands are labeled with an asterisk.

*Arabidopsis thaliana *encodes seven GPx, of which three are organellar targeted by Nterminal transit peptides [17,66]. GPx6 (At4g11600) is suggested to be alternatively targeted to mitochondria and the cytosol, while GPx1 (At2g25080) and Gpx7 (At4g31870) are chloroplast-targeted and protect plants from photooxidative stress [22]. The conserved exonintron-structure strongly indicates a common origin of GPx1 and GPx7 (Fig. 15), as well as of GPx6 (data not shown). Superimposure of AtGPx1 and AtGPx7 proteins modeled and presented by SWISS-MODEL and Swiss-pdbViewer [67] showed highly similar structures (data not shown). In contrast, AtGPx6 differs structurally by replacement of helix 2 by a not structured protein domain and a shorter helix 1 (Fig. 16A).

#### Selaginella chloroplast GPx

In the genome of the two haplotypes of *Selaginella moellendorffii *three different loci (SmGPxA, SmGPxB and SmGPxC) were observed, which encode GPx similar to Arabidopsis GPx1 and GPx7. To date these six peroxidase sequences are not covered by PeroxiBase. SmGPxA and SmGPxB proteins show N-terminal extensions similar to organellar targeting signals. For SmGPxA, both, the TargetP- and ATP-values were high (Table 1) and indicate chloroplast targeting. For the two SmGPxB isoforms the ATP-values were high, but the TargetP-values low (Table 1). An 18 amino acids long N-terminal α-helix (A_5 _- W_22_) with several hydroxylated amino acids and positive charges, however, points to organellar targeting and recognition by the protein import complex.

BLASTN-searches demonstrated strongly different expression intensities for the homologous GPx genes in the two Selaginella haplotypes, for which similar expression intensities of APx and Prx genes were shown (Table 1). While 8 ESTs were observed for SmGPxB.1, none was found for SmGPxB.2.

An additional gene with putative GPx function, SmGPxC, was detected in the genome of both analyzed haplotypes of Selaginella (Table 1). Lack of an N-terminal chloroplast targeting signal (Fig. 14A) suggests extraplastidic localization.

Comparison of the modeled protein structures revealed almost structural identity of SmGPxA.1 and SmGPxA.2 to AtGPx1 and AtGPx7. The fold of SmGPxB proteins differed. Superimposure of SmGPxB proteins and AtGPx1 demonstrates a less structured organization of amino acid 175 to 186, which form an α-helix in SmGPxA and the Arabidopsis chloroplast GPx protein (Fig. 15B). Due to the unstructured domain, SmGPxB shows higher similarity to mitochondrial AtGPx6 (data not shown). For both SmGPxA and SmGPxB haploforms cDNA fragments of the predicted size (SmGPxA.1: 417 bp; SmGPxA.2: 415 bp; SmGPxB.1: 298 bp and SmGPxB.2: 296 bp) could be amplified by cDNA-specific saturating RT-PCR (Fig. 16) demonstrating expressional activity of all predicted Selaginella genes encoding chloroplast GPx.

#### Physcomitrella chloroplast GPx

In Physcomitrella two GPx genes encoding putative organellar targeting signals with high ATP- and TargetP-scores (Table 1) were identified. The exon-intron-structures of PpGPxA and PpGPxB are conserved with Arabidopsis and Selaginella homologs (Fig. 15). Similar to APx and Prx genes, in Physcomitrella the introns are longer than in Arabidopsis and much longer than in Selaginella.

For PpGPxA 11 ESTs were found additionally demonstrating expressional activity. One of the four ESTs covering the 3'-end of the ORF indicates alternative splicing of the last intron. The last intron is maintained in this EST, while it is spliced out in three other ESTs (for PpGPxA.1), like in all Arabidopsis and Selaginella ESTs. The single non-matching EST shows that Physcomitrella has a small propensity to encode C-terminal aberrant GPx (PpGPxA.2) (Fig. 13 and 14; Table 1). For the second PpGPx locus, PpGPxB, no EST was found in the data base (Table 1).

To test for gene expression activity saturating RT-PCR was performed with RNA isolated from sterile grown gametophytes. The reaction gave a strong signal of the expected size for PpGPxA.1 demonstrating presence of PpGPxA.1 mRNA. For the splice variant PpGPxA.2 PCR with variant-specific primer combinations gave a weak band of 361 bp besides two larger, non-specific bands. In contrast, expressional activity of PpGPxB could not been shown by RT-PCR. Under the applied temperature and MgCl_2_-conditions only DNA fragments were amplified, which were too large to represent PpGPxB (Fig. 16).

**Figure 17:**
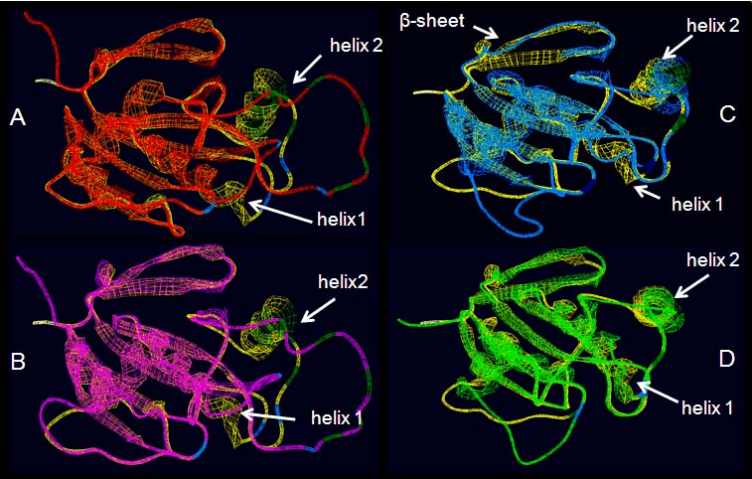
**Superimposition of AtGPx1 (yellow) and (A) AtGPx6 (red), (B) SmGPxB**.1 (pink), (C) PpGPxB (light blue) and (D) CrGPxC (green). The catalytic sites are labeled in blue. The arrows point at the helices 1 and 2 for which the structures of the presented proteins differ from the AtGPx1/AtGPx7 structure.

Strong sequence modifications in the domains encoding the otherwise highly conserved FPCNQFxxQEP environment [65] of the catalytic site (label "C" in Fig. 14A; e.g. Q^167^T) in addition to several substitutions (e.g. Y/H^105^R and V/I^117^T) and the untypical C-terminal extension (Fig. 14A) suggest that PpGPxB, if expressed, would have various structural modifications (Fig. 17C). Therefore, it is very likely that this protein is not fully functional. Superimposure of protein models demonstrated high structural similarity of PpGPxA.1 and PpGPxA.2 to AtGPx1 and AtGPx7 (data not shown) indicating that PpGPxA is the Physcomitrella homolog of Arabidopsis GPx1 and GPx7.

#### Chlamydomonas chloroplast GPx

Consistent with the previous analysis by Dayer et al. [16], three organellar GPx were detected in the genome of *Chlamydomonas reinhardtii *(Table 1). Two of them CrGPxA (designated GPx1 by [16]) and CrGPxB (GPx2 in [16]) are selenoproteins (X^137^) as GPx are in many animals and microbia [68]. On the contrary, CrGPxC (GPx5 in [16]) is a nonselenocysteine GPx (C^136^) like the GPx identified in the other plant analyzed species (Fig. 14A). Our data are widely consistent with that of Dayer et al. [16], except for the primary sequence of CrGPxA. For this protein the new translation demonstrated that in the previous description [16] a G (G^137 ^in Fig. 14A) was missing at the position following the selenocysteine.

The gene structures of CrGPx genes are hardly conserved (Fig. 15). Except the splice site at the end of exon1 of CrGPxC (135-2), Chlamydomonas GPx transcripts and GPx hnRNA from the other tested species have no splice site in common (Fig. 15).

Expressional activity of CrGPxB and CrGPxC was confirmed by saturating RT-PCR (Fig. 16). Under a wide range of conditions (annealing temperature: 46 - 60°C and 1.5 - 3 mM MgCl_2_), no signals were observed which may indicate expression of CrGPxA in our plant material. Expression of CrGPxA is either very low or restricted to very specific conditions.

The modeled two selenocysteine isoforms CrGPxA and CrGPxB show structural similarity to AtGPx1 and AtGPx7 (data not shown). The predicted structure of the well expressed nonselenocysteine-type CrGPxC shows several structural aberrations if compared to AtGPx1 (Fig. 17D), indicating a distinct type of GPx. Since even the position of the active site (labeled blue in fig. 17D) is affected by aberrant twists in helix 1 and helix 2, differences in the catalytic activity have to be assumed. Despite the strong differences to AtGPx1, comparison to human GPx (data not shown), showed stronger structural similarity of CrGPxC to monomeric human GPx4 than to tetrameric GPx1 (data not shown), demonstrating that the less conserved Chlamydomonas isoforms also belongs to the monomeric GPx cluster.

#### Phylogenetic tree analysis of plant GPx

According to Margis et al. [17], plant GPx evolved by four major gene duplication events from a single ancestral gene. The initial duplication has been supposed to have taken place prior to separation of monocots and dicots or even before separation of gymnosperms and angiosperms. Phylogenetic analysis was performed with the protein sequences of the here investigated genes and sequences taken from PeroxiBase using neighborhood joining (Fig. 14B), maximum parsimony and minimum evolution algorithms (Additional files 9 and 10). For *Arabidopsis thaliana *all GPx were included in this analyses, irrespective of the subcellular localization of the enzymes. The distance of the chloroplast isoforms to the other Arabidopsis GPx demonstrates that chloroplast AtGPx1 and AtGPx7 result from a late gene duplication which took place after separation of mitochondrial, cytoplasmic and chloroplast paralogs. For the chloroplast GPx genes from the other species a more ancient separation can be assumed from the distant positions in the calculated phylogenetic trees (Fig. 14B; Additional files 9 and 10). The variability between the three different types of phylogenetic trees makes it impossible to definitely answer the question whether chloroplast targeting was established independently for different chloroplast paralogs or prior to the final gene duplication event. The phylogenetic trees, however, suggest early separation and independent evolution of GPx isoforms. Therefore, compared to Margis' et al. [17] assumptions, our analysis shows that the gene duplication resulting in chloroplast paralogs occurred much earlier than the angiopserm - gymnosperm separation.

### Relative expression of peroxiredoxin, ascorbate peroxidase and glutathione peroxidase genes

Microarray-based data (available from TAIR) show that sAPx and tAPx genes are weakly expressed in Arabidopsis compared to 2CP (expression level of the entire rosette after transition to flowering: sAPx: 328.86; tAPx: 403.91; 2CPA: 3184.8; 2CPB 1222.76). For the APx encoded by the two Selaginella haplotypes, 3 and 6 sAPx ESTs and 9 and 10 tAPx ESTs (Table 1) were identified in the EST cluster analysis. In the same data set 67 and 60 ESTs for Sm2CPA, 12 and 15 for SmPrxII and 21 and 7 for SmPrxQ were counted. However, EST numbers are not precise measures for gene expression activities, the relative numbers indicate a rather weak expression of APx if compared to Prx. For *P. patens*, the relative EST counts indicate stronger expression of PptAPx. Within the group of Prx, the Pp2CPA gene was slightly less covered by ESTs than the genes for PpPrxIIA and PpPrxIIB together, but more than the three PrxQ genes together. In *C. reinhardtii*, however, for CrsAPxA only 5 ESTs were counted in the JGI database, Cr2CPA expression was represented by 121 ESTs. The other Prx genes and the GPx genes were much less active, indicating that Cr2CPA has the strongest impact on the chloroplast antioxidant system.

In general, EST counts were lowest for GPx (Table 1). In combination with functional restrictions indicated by superimposure of the predicted amino acid sequences (Fig. 17A) the analysis indicates a subordinary or very specific function of GPx in chloroplasts. In the comparison of species, *Selaginella moellendorffii *may be excluded from this general conclusion. With 26 SmGPxA ESTs for each haplotype (compared to 3 + 6 sAPx, 9 + 10 tAPx) the EST counts demonstrate similar or slightly stronger expression of GPx than APx genes.

## Discussion

In the antioxidant defense against photooxidative ROS formation, APx and GPx as well as Prx provide protection against peroxides and, therefore, have partially overlapping functions [22,23,31]. Despite the long time of evolution following the separation of chlorophytes, mosses, ferns and higher plants, *Chlamydomonas reinhardtii*, *Physcomitrella patens*, *Selaginella moellendorffii *and *Arabidopsis thaliana *maintained parallel expression of chloroplast APx and GPx and all of them also target three types of Prx to chloroplasts (Table 1).

The EST-assembly and sequence homology based genome wide search for ORFs demonstrated that the gene copy number and the relative expression intensity changed during evolution: In *Selaginella moellendorffii *and in *Physcomitrella patens *the genes for chloroplast PrxII and PrxQ were duplicated and triplicated, respectively. In Selaginella, EST counts showed a much stronger GPx expression relative to APx expression than in Arabidopsis. Together with amplification of PrxII and PrxQ gene copy numbers (Table 1), this data indicate a stronger preference for the non-ascorbate-linked antioxidant system in the spikemoss. In *Physcomitrella patens*, the chloroplast antioxidant system lacks stromal APx activity. *Chlamydomonas reinhardtii *does not have thylakoid-bound APx, but encodes three chloroplast-targeted GPx. One of them is typical for plants [12] and two are two selenocysteine-type GPx, which are otherwise typical for animals and microbia [50,68]. This comparison suggests that the dominance of APx in the chloroplast antioxidant system [11] is not ubiquitous in the plant kingdom and may mainly be characteristic for higher land plants, for which it was first described [2].

### Chloroplast ascorbate peroxidase genes share a common evolutionary origin

Similarities in the amino acid composition (Fig. 1A) and in the gene structures (Fig. 2) demonstrate that Chlamydomonas, Selaginella and Arabidopsis APx genes are related. The ancestral gene dates back to times before chlorophytes and streptophytes split into two lineages. Besides CrsAPxA, which was already annotated as APx in the NCBI-database [50], the chlorophyte Chlamydomonas has a second gene, CrsAPxB, encoding a chloroplast ascorbate peroxidase (Fig. 1) [69]. Due to specific sequence characteristics CrsAPxB is annotated as putative bifunctional ascorbate-cytochrome c peroxidase in PeroxiBase. The protein shows highest similarity to proteins encoded by red algae, but no CrsAPxB ortholog has been observed in any streptophyte. Since the deduced amino acid sequence showed various modifications in heme and substrate coordination sites (Fig. 1A), CrsAPxB is very likely a non-functional pseudogene.

### CrsAPxA has an extra-long regulatory loop between F/W^288 ^and W^327^

Compared to the other chloroplast APx (summarized in PeroxiBase), CrsAPxA shows two short loops on the protein surface (Fig. 4). EVaLoII is placed between F/W^288 ^and W^327^. As shown by Kitajima et al. [70] and Teixeira et al. [23], the distance between this positions is extended in all chloroplast APx if compared to cytosolic and microbody-localized APx. The loop increases the susceptibility of the heme to H_2_O_2 _[49]. In CrsAPxA this loop is further extended by 19 amino acids (Fig. 1A). Since for *Chlamydomonas sp*. W80 a chloroplast APx, which shows a chloroplast-typical loop, was reported to be stable in the presence of excess H_2_O_2 _in an ascorbate-depleted medium [71-73], a larger loop, such as EVaLoII, might be necessary for increasing the H_2_O_2 _sensitivity and enable flood gate control between H_2_O_2 _detoxification and H_2_O_2 _signaling inside chloroplasts [23,43,72].

### In Physcomitrella, the ancestral APx is replaced by an APx of retrotransposonal origin

*Physcomitrella patens *encodes only a tAPx, but no sAPx (Fig. 2). The Physcomitrella gene for the chloroplast APx has a single atypical splice site and is flanked by Angela LTR retrotransponson footprints indicating a retrotransposonal origin. For Physcomitrella several copia-type elements have been inserted into the genome in waves. On average, insertions took place every 3.9 million years [74]. Mosses separated from tracheophytes, such as ferns and seed plants, 360 - 380 million years ago. However it is not possible to precisely date retrotransposition events, the insertion may have taken place independently in the moss branch. Due to the unconserved splice site (if compared to the other chloroplast APx genes analyzed), it is tempting to assume that the intron was inserted after the retro-transposition event and does not result from incomplete splicing of a retrotranscribed hnRNA.

In neighborhood joining (Fig. 1B), minimal evolution and maximum parsimony trees (Additional files 1 and 2) calculated from ClustalW protein alignment of all identified and all in PeroxiBase listed putatively full-length plant ascorbate peroxidases, the PptAPx clusters with chloroplast APx (between chloroplast APx from seed plants and non-seed plants) suggesting that it originates from a chloroplast APx encoding mRNA.

Unlike other chloroplast APx, in PptAPx at position 282 an arginine residue is substituted by a histidine residue. H^282 ^corresponds to H^169 ^in cytosolic APx of pea. The histidine side chain forms a salt bridge with the propionated side chain of the heme [75]. The H^282 ^is typical for cytosolic APx and prevents H_2_O_2_-dependent decomposition of compound I in the absence of ascorbate [71] suggesting that PptAPx is less H_2_O_2_-sensitive than other chloroplast APx.

### Physcomitrella lacks sAPx function and substituted it by Prx gene multiplication

The single Physcomitrella plastid APx gene encodes a tAPx with a C-terminal transmembrane anchoring helix. Unlike tobacco, spinach and pumpkin APx genes [20,76,77], EST-analysis gave no indication for alternative splicing of the hnRNA into transcripts encoding thylakoid and stromal isoforms (data not shown). It is concluded that *Physcomitrella patens *lacks sAPx function. Studies in transgenic Arabidopsis demonstrated that sAPx has a stronger protective function under photooxidative stress conditions than tAPx [31]. Consequently, lack of sAPx activity may limit chloroplast antioxidant protection in Physcomitrella. In Arabidopsis, Prx expression increases in response to insufficient chloroplast APx activity [31].

From the analysis of today's genome structures, it is not possible to predict whether Physcomitrella has lost the ancestral APx gene prior or post to maturation of the retrotransposonal gene. The natural habitat of the moss is shady. Considering the low-light environment, the phylogenetic ancestors of Physcomitrella may have lost its ancestral chloroplast APx gene even before the novel gene was functionally adapted by evolution of suitable promoter elements and chloroplast targeting signals. In Physcomitrella, the genes for chloroplast Prx have been multiplied. Compared to two 2CP, one PrxII and one PrxQ in *Arabidopsis thaliana*, *Physcomitrella patens *expresses two chloroplast 2CP, two chloroplast PrxII and three chloroplast PrxQ paralogs (Table 1).

### 2-Cys peroxiredoxins evolved independently in streptophytes and chlorophytes

2-Cys peroxiredoxins are nuclear encoded chloroplast enzymes in higher plants [21], but cytosolic enzymes in heterotrophic eukaryotes [78]. Based on sequence comparison with cyanobacterial 2CP and plastome encoded 2CP from the rhodobiont *Porphyra purpurea*, it has been postulated that higher plant 2CP are of endosymbiotic origin [21]. The gene structures (Fig. 6) and sequence characteristics (Fig. 5A) demonstrated that 2CP of all analyzed streptophytes are of common origin. On the contrary, none of the 2CP genes of the chlorophyte *Chlamydomonas reinhardtii *showed a conserved splice site and, thus, might have evolved independently.

In Arabidopsis, the gene structures (Fig. 6) and the deduced amino acid sequences (Fig. 5A) are more similar to each other than to the two 2CP genes of Selaginella indicating distinct gene duplication events. Since no ESTs were observed for Sm2CPB, the gene could be a silent pseudogene. It might have started to accumulate mutations due to lack of selective pressure on the functional activity of the Sm2CPB encoding gene. This hypothesis is supported by changes in otherwise conserved charged amino acid residues (e.g. D^148^N, D^156^H, D/E^166^N, D^181^N, E^201^Q and S^289^N), a deletion in the conserved KEY-motif [79] and the elongated N-terminus of mature Sm2CPB (aa^101 ^- aa^138^) (Fig. 5A). These modifications change the charge of the protein surface and very likely disturb protein dimerization, which is important for formation of intermolecular disulfide bonds during the reaction cycle.

### There has been a strong selective pressure on stromal Prx activity

Studies of 2CP antisense lines demonstrated that decreased 2CP activity can be compensated by increased induction of APx gene expression [80]. Chloroplast APx has a by three to four magnitudes higher peroxidative activity than Prx [81]. Thus, slightly higher expression of APx could support the antioxidant protection of plants similar to a strong induction of Prx. However, sequence and structural conservation (Fig. 5, 8, 9 and 15) reports that there has been a strong selective pressure on the maintenance of Prx, especially on 2CP, during evolution of plants. As estimated from array hybridizations and EST-counts, the relative expression of 2CP is higher in Arabidopsis, Selaginella and Chlamydomonas than APx expression. For Physcomitrella, difficulties with scaffold arrangement in case of the Pp2CPB gene http://www.cosmoss.org limited EST sorting. One reason, which accounts for maintenance of Prx could be, that they can reduce a wide range of alkyl hydroperoxides [30], while APx isoforms are almost specific for H_2_O_2 _[4]. However, since GPx also detoxify alkyl hydroperoxides, Prx could have been replaced by GPx.

Another reason for keeping Prx activity could be a better antioxidant protection in post-stress phases: Upon severe oxidative stress APx and Prx are inactivated [70]. In APx, W^140 ^is cross-linked to the heme by excess H_2_O_2 _[81]. In Prx the peroxidatic C is sulfinylated [30]. Sulfinylated 2CP can be regenerated by e.g. sulfiredoxins [54], while chloroplast APx are irreversibly inhibited [81]. This re-activation option gives 2CP a special importance during post-stress acclimation and may explain why all plants maintained Prx genes during evolution. In higher plants, 2CP stability against oxidative damage is supported by accumulation of high protein amounts [30]. 2CP is assumed to be an ancient, stable peroxidase which is expressed prior to activation of APx in young tissues [82].

### Chlamydomonas weakly expresses a putative cytosolic 2CP

In Chlamydomonas proteins, the chloroplast targeting signals are much shorter and less hydroxylated and positively charged than targeting signals from the other enzymes analyzed (Fig. 1A, 5A, 8A, 9A and 14A). On the one hand this impedes targeting predictions, on the other hand, in Chlamydomonas the single large chloroplast covers almost 90% of the cell lumen. A recent study [41] demonstrated that protein import is controlled by mRNA availability and localized translation.

In the chlorophyte Chlamydomonas three 2CP genes were observed. Their gene structure is not conserved (Fig. 6). Compared to the other 2CP analyzed in this study, the amino acid sequences of the proteins are more variable (Fig. 5A). Especially in Cr2CPB, the C-terminus is highly charged and modified in the KEY-motif (aa^330 ^- aa^332^) [56]. In the C-terminus, the protein has three prolin residues, compared to four prolins in other plant 2CP and two prolins in non-plant 2CP [18]. Taken this together with the modified charge pattern of the Cterminus, Cr2CPB resembles non-plant 2CP more than plant 2CP according to the criteria summarized by König et al. [18].

Chlorophytes have diverged from streptophytes more than 1 billion years ago [83]. The chlorobionts share common ancestors with rhodobionts of which some, e.g. *Porphyra purpurea*, encode 2CP in their plastomes [21]. Many non-photosynthetic eukaryotes, including mammals and fungi, express cytosolic 2CP [21]. Maximum parsimony analysis of amino acid similarity (Additional file 4) indicates that the three Chlamydomonas 2CP are related. Amino acid sequence characteristics (Fig. 5A) and, especially, gene structure analysis (Fig. 6) suggest that the chlorophyte either retained a cytosolic 2CP of different evolutionary origin than the chloroplast 2CP of higher plants or indicate a chloroplast 2CP with an plastid-atypical amino acid sequence.

### Gene structure evolution of PrxII and PrxQ

Chloroplast PrxII are encoded by single exon genes in streptophytes, but by a five exon gene in Chlamydomonas. Despite the difference in the gene structure (Fig. 11), they show high amino acid sequence conservation between the species (Fig. 9A and 9B). From comparison of APx, 2CP and PrxQ genes, it can be concluded that most intron insertions may have taken place prior to divergence of mosses, ferns and seed plants. However, sequence analysis provides no explanation why so many intron-insertions took place in the gene for chloroplast PrxII of Chlamydomonas or intron deletions in the streptophytes. In the streptophyte branch, consistent with genome wide comparisons [84], the gene structures encoding the mature parts of PrxQ and APx are conserved between the analyzed species. Only the intron lengths are variable in these genes. Based on comparison of Arabidopsis and rice genomes Roy and Penny [85] concluded that the main difference in intron numbers results from mRNA-mediated intron loss. Accordingly, it is more likely that the streptophytes lost introns than that they were inserted in the chlorophyte genes.

### Thylakoid localization of PrxQ might be streptophyte specific

In *Arabidopsis thaliana*, PrxQ is post-translationally targeted to the thylakoid lumen [19]. Protein trafficking is controlled by bi-partite transit peptides [28]. Comparison of the AtPrxQ transit peptide with the transit peptides of the other streptophyte PrxQ shows similar lengths and distribution of positively charged and hydroxylated amino acid residues (Fig. 8A). In contrast, the transite peptide of CrPrxQ is much shorter and stronger positively charged in its second half (aa^71^-aa^90^) indicating that the thylakoid lumen address is missing and the protein is targeted to the chloroplast stroma [28]. Furthermore, CrPrxQ lacks negative charges at position 118, 119, 135 and positive charges at position 138, 139, 156, 175 and 200. A high number of positive charges in the mature protein is characteristic for streptophyte PrxQ (Fig. 8A). They can be protonated upon thylakoid lumen acidification and regulate enzyme activity. Several of these residues are not conserved in CrPrxQ (Fig. 8A) suggesting a different regulation and, since the short transit peptide also gives no indication for a thylakoid-lumen targeting signal, also a different localization.

### Chloroplast glutathione peroxidase genes are highly conserved in streptophytes, but only weakly related to chlorophyte GPx

Compared to the other investigated types of chloroplast antioxidant enzymes, the gene structure is most conserved for GPx in the streptophyte branch of the plant kingdom (Fig. 15). In genes from Physcomitrella, Selaginella and Arabidopsis all splice sites were absolutely conserved in the gene part encoding the mature protein. In Physcomitrella one identified EST may show alternative splicing of the last intron. The high conservation and the high similarity of the protein primary sequences demonstrate a strong evolutionary pressure on maintenance of GPx function. In Selaginella, one pseudogene with modified gene structure and many substitutions, SmGPxD, was observed (genome location 123: 187057 - 188973; data not shown). Since this gene was only found in one of the two sequenced haplotypes, it may result from a late, haplotype specific gene duplication. If this was the case, it would give additional stress on the hypothesis of the high evolutionary pressure to conserve gene structures of ancient genes. The fact that an extreme extention of intron1 is accompanied by very low transcript coverage of PpGPxB, may support the importance of GPx gene structure conservation, such as structural organization of the pre-peptide encoding exon.

### Evolution of GPx genes included gene duplication and alternative splicing

It is generally assumed that gene duplication increased the gene copy number of GPx in plants [17]. Concerning chloroplast isoforms, phylogenetic tree analysis (Fig. 14B; Additional files 9 and 10) and comparison of amino acid residues (Fig. 14A) gave surprisingly little information on the general relation of the GPx genes. The several chloroplast GPx proteins clustered with different proteins in maximum parsimony, minimum evolution and neighborhood joining trees (Fig. 14B; Additional files 9 and 10). However, gene structure analysis (Fig. 15) indicates a common origin of streptophyte GPx. Weak similarities in the exon-intron-structure between Chlamydomonas and streptophyte GPx and co-existence of selenocysteine- and cysteine-type GPx in Chlamydomonas, but not in streptophytes, demonstrates parallel independent evolution of GPx branches in chlorobionts.

In contrast to early chloroplast GPx evolution, conclusions can be drawn for the latest GPx duplication: Since only single genes for chloroplast isoforms were observed in Selaginella (per haplotype) and Physcomitrella, duplication of the common ancestor gene into AtGPx1 and AtGPx7 may have occurred after separation of phanerophytes and seed plants. In all phylogenetic trees calculated from ClustalW alignments, a single Cycas protein (5756 in Peroxibase) is the closest relative of AtGPx1 and AtGPx7. Without any paralogs in Cycas, the final duplication may even have taken place after separation of gymnosperms and angiosperms.

In the minimum evolution and neighborhood joining trees (Fig. 14B, Additional files 9 and 10), the most similar relatives of AtGPx1 and AtGPx7 are grass proteins (3059 and 5740 in Peroxibase). The other grass proteins included in the phylogenetic analysis are more similar to mitochondrial AtGPx6, indicating that separation of the AtGPx6 lineage and the AtGPx1/AtGPx7 branch took place prior to separation of monocots and dicots. Since the AtGPx1/AtGPx7-cluster compromises only spermatophyte proteins, suppgests that separation of the mitochondrial isoforms is a late event.

According to neighborhood joining analysis (Fig. 14B), the Arabidopsis chloroplast proteins cluster with proteins encoded by basal gymnosperms (Cycas; Peroxibase 4303), basal angiosperms (Amborella; Peroxibase 4302) and liverworts (Marchantia; Peroxibase 5757). This result demonstrates that the Arabidopsis chloroplast GPx proteins have been more conserved over time and are, presumably, more ancient than the mitochondrial AtGPx6 ancestor. GPx6-like proteins clusters exclusively with grass proteins and, consequently, may be spermatophyte-specific.

Protein modeling clearly separates AtGPx6 from AtGPx1 and AtGPx7 (Fig. 17A), although the overall gene structures are still very similar (data not shown). In Selaginella, the predicted three-dimensional structure of SmGPxA protein is, consistent with neighborhood joining weighted primary sequence analysis, similar to AtGPx1/AtGPx7. On the contrary, SmGPxB shows stronger structural similarity to AtGPx6 indicating that the two newly described organellar Selaginella GPx are homologues or AtGPx1/AtGPx7 and AtGPx6, respectively.

The most diverse GPx-types were observed in the green algae Chlamydomonas (Fig. 14A, Fig. 14B and 15, additional files 9 and 10). The cysteine-type GPx of Chlamydomonas (CrGPxC) has a conserved splice site with the other analyzed plant GPx between exon1 and exon2, suggesting an evolutionary relation to streptophyte GPx. The gene structures of the selenocysteine-type GPx are non-conserved. Comparison of the protein structure with human seleno-GPx demonstrated that the structure is more similar to monomeric human GPx4 than to tetrameric human GPx1 (data not shown). Consistent with Margis et al. [17], we therefore conclude that all plant GPx have evolved from a common ancestor. In Chlamydomonas low sequence and gene structure similarity between the three putative organellar GPx genes reflects high diversification. In streptophytes only one branch was maintained as indicated by common exon-intron structures. Additional gene duplications, such as in case of AtGPx6 separation from the AtGPx1/AtGPx7 ancestral gene, and introduction of alternative splicing, such as in the last exon of PpGPxA, resulted in further gene diversification.

## Conclusions

The study demonstrates that, irrespective of how much time was available for modifications of intron lengths and individual amino acid residues, there had been an evolutionary pressure on maintenance of all three chloroplast peroxide detoxification systems, namely APx, GPx and Prx. Especially adaptation of an APx retrotransposon in Physcomitrella for post-translational protein import into chloroplasts and multiplication of PrxQ and PrxII genes show the strong evolutionary pressure plants have perceived on having APx, GPx and Prx controlled chloroplast antioxidant protection in parallel. Gene structures demonstrate that streptophyte and chlorophyte APx genes evolved from a common ancestral gene. The structures of the Prx genes, in contrast, suggest at least a partially independent evolution, which resulted in putatively non-plastidic or atypical 2CP isoforms, a potentially non-thylakoid lumen PrxQ and a PrxII gene with three chlorophyte-specific introns in Chlamydomonas. In addition, streptophytes and chlorophytes differ in their GPx patterns, with Chlamydomonas expressing two seleno-GPx and one cysteine-type GPx, while only cysteine GPx are encoded in streptophytes. The comparison of model chlorobiont genomes demonstrated that the complexity and the individual components of the chloroplast antioxidant system were targets for mutations and selection. In summary, this comparison provides a new basis for analyzing the function of individual components of the chloroplast antioxidant enzyme defense with respect to plant tolerance against photooxidative stress.

## Methods

### Basic data mining

TBLASTX searches (BLOSUM62; threshold: 10; word size: 3) were performed with the coding sequences of *Arabidopsis thaliana *stromal and thylakoid ascorbate peroxidases (At4g08390 and At1g77490), GPx1 (At2g25080), GPx7 (At4g31870) and chloroplast peroxiredoxins (2CPA: At3g11630; 2CPB: At5g06290; PrxQ: At3g26060; PrxIIE: At3g52960) [29] in EST-databases of *Chlamydomonas reinhardtii *[ChlamyDB http://www.chlamy.org/ and JGI http://genome.jgi-psf.org], *Physcomitrella patens *[cosmoss http://www.cosmoss.org/] and *Selaginella moellendorfii *[JGI http://genome.jgipsf.org/Selmo1/Selmo1.home.html]. The collected APx, GPx and Prx ESTs were clustered based on sequence similarity using the DNA Identity Matrix/Unity matrix of ClustalW2.0 http://www.ebi.ac.uk/Tools/clustalw2[32], which is a fast and sufficient tool to distinguish between the expected perfect matches and isogenes-dependent variations. The consensus cDNA sequences were translated into an amino acid sequence using online resources and the standard eukaryotic gene code http://bio.lundberg.gu.se/edu/translat.html.

The deduced amino acid sequences were aligned with Arabidopsis APx, GPx or Prx using CLUSTALW2.0 to test the sequences for conserved motifs. With the 200 bp ends of the consensus sequences the data bases were searched using the BLASTN algorithm (threshold: 10; word size: 28; match/mismatch scores: 1/-2) for ESTs covering the less conserved 5'- and 3'-ends.

For still incomplete or potentially incomplete cDNAs, 2000 bp upstream of the 5'-end of the cDNA the respective genomic DNA was screened for transcription start sites using DBTSS [33], for 5'-exons using FEX [34] via the Softberry-interface http://linux1.softberry.com, BLAST searches in the EMBL Plant EST database and, finally, by hand, for putative S, T and R rich peptides and other sequence characteristics of chloroplast targeting signals as summarized in [86].

Finally, PeroxiBase http://peroxibase.isb-sib.ch/search.php[42] was screened for putative chloroplast peroxidases as described in the results section. For the final phylogenetic analysis also cyanobacterial PrxQ and 2CP and extra-organellar and mitochondrial APx, GPx and PrxII were retrieved from PeroxiBase for comparison. All collected amino acid sequences were aligned using CLUSTALW2.0 [32]. For calculation of phylogenetic trees, incomplete sequences were removed from the analysis.

### Discriminating between putative chloroplast and non-chloroplast isoforms

To distinguish between chloroplast and non-chloroplast isoforms, the protein sequences with putative N-terminal extensions were analyzed by TargetP [38] and ATP (http://www.cosmoss.org/bm/ATP; [32]) for their chloroplast targeting probability.

### Determination of EST counts

The number of ESTs was used as a relative measure for the expression intensity. To identify all ESTs, the full length cDNA sequences were used as queries for BLASTN searches (threshold: 10; word size: 28; match/mismatch scores: 1/-2) in the respective EST database [ChlamyDB http://www.chlamy.org/ and JGI http://genome.jgi-psf.org for Chlamydomonas, Cosmoss http://www.cosmoss.org/ for Physcomitrella and JGI http://genome.jgi-psf.org/Selmo1/Selmo1.home.html for Selaginella] and the EMBL Plant EST database. The ESTs were counted for each non-identical EST cluster.

### Calculation of GC-contents

The GC-contents were calculated based on the codon usage statistics tool CHIPS via the Mobyle-webpage http://mobyle.pasteur.fr.

### Prediction of the gene models

The EST-assembly-based consensus sequences were aligned to genomic DNA by BLASTN [29] and ClustalW2.0 [32] to identify exons. In case of Selaginella, genomic data and EST data are combined from two mixed haplotypes [46,87]. To distinguish between alleles and genes, in the gene structures of the respective genomic scaffolds were compared with computational gene models provided by JGI http://genome.jgipsf.org/Selmo1/Selmo1.home.html. If the gene environment was similar the two found gene models were designated as alleles, otherwise as isogenes.

### Comparison of splice sites

The positions of splice sites were compared relative to the amino acid positions in the protein alignments depicted in fig. 1A, 5A, 8A, 9A and 14A. The splice position within the respective codon was designated 0, 1, and 2 according to [88].

### Protein alignments and protein comparison

The amino acid sequences of proteins were aligned by using the EMBL-EBI tool MUSCLE [89] and ClustalW2.0 [32], and by MEGA 4.1. The phylogenetic trees were calculated based on ClustalW2.0 alignments using the Mega 4.1 software package [90]. Data retrieved for neighborhood joining were compared with minimum evolution and maximum parsimony methods. The quality of the predicted trees was tested by calculating the bootstrap values based on 500 replicates. In the sequence comparisons, amino acid substitutions were weighted according to the Poisson calculation and the PAM250 matrix.

### Secondary structure prediction and 3D-structure modeling

Secondary structure analyses were performed with PredictProtein [35]. Transmembrane helices and their lipophilicity were predicted using TMHMM [36], TMPro [37] and WHEEL http://cti.itc.virginia.edu/~cmg/Demo/wheel/wheel_instructions.html. AmphipaSeeK [91] was used to search for putative in-plane membrane anchors. In addition, the tool was utilized to confirm the PredictProtein-based protein structure analysis. The 3 D structures were predicted by using the freely available alignment-based modeling tool SWISS-MODEL and the SwisspdbViewer DeepView 4.0 [67].

### Plant growth conditions

*Chlamydomonas reinhardtii *was grown at 12 - 20 Zmol m^-2 ^s^-1 ^and 12 h light/12 h dark under sterile conditions in TAP liquid media and at 80-1000 μmol m^-2 ^s^-1 ^and 12 h light/12 h dark on TAP agar plates [92], *Physcomitrella patens ssp. patens gametophytes *were grown at 70 - 80 μmol m^-2 ^s^-^1 and 14 h light/10 h dark on modified Knop-medium according to Bopp and Brandes [93] under sterile conditions and *Selaginella moellendorffii *sporophytes were grown in the green house on soil and 14 h per day illumination with 50 μmol quanta m^-2 ^s^-1 ^white light. *Arabidopsis thaliana *was cultivated for 6 weeks on soil in a growth chamber at 20°C and illuminated with 80 -100 μmol m^-2 ^s^-1 ^for 10 h per day.

### Isolation of genomic DNA

For isolation of genomic DNA 100 - 150 mg Selaginella sporophytes or Physcomitrella gametophytes were ground to a fine powder in liquid nitrogen and extracted for 30 min at 60°C with 1 ml CTAB medium (100 mM Tris-HCl, pH 8.0; 1,4 M NaCl, 20 mM EDTA and 2% (w/v) CTAB). The extract was subsequently extracted with 1 ml chloroform. The DNA was precipitated from the aqueous phase with 1/10 volume 3 M Na-acetate (pH 5.2) and 0.8 volumes isopropanol during a 15 min incubation period at room temperature, washed in 70% (v/v) ethanol and dissolved in sterile H_2_O.

### RNA extraction, RT-PCR, PCR-amplification of genomic DNA and analysis

Chlamydomonas RNA was extracted from 100 mg dry algae pellets as described in Heiber et al. [94] for ground Arabidopsis plant material. Physcomitrella and Selaginella RNAs were isolated from 100 mg plant material with RNeasy Mini Kit (Qiagen, Hilden, Germany) according to the manufactures recommendations. Reverse transcription was performed according to Heiber et al. [94]. In the PCR reactions with the primers CrsAPxA-S: GTTGAGCAGCTGAAGGCG, CrsAPxA-A: CTCAGTCCAGAGTAACGGGC, CrsAPxB-S: CCCGGCGACTACGCG, CrsAPxB-A: CAACTACCGTACTGCTGCAACG, PptAPx-S: GCCATAGCCTCTGATCC, PptAPx-A: GACATTGTTAAATAAACTAGCCAAG, SmtAPx-S: GCGAATGATCTCGAAGAAG; SmtAPx-A: CTAAAACCCACCGAATAGCC, SmsAPx-S: CTCGATCAGCTAGTGGGA, SmsAPx-A: CTAACTTGTGCTCTCGTCGAT, CrGPxA-S: CCTAACACCTGTGCTAGCTTC; CrGPxA-A: CGACAATGTACTTCTCGAGC; CrGPxB-S: GACTCCATCTACCAGTTCAG; CrGPxB-A: CGTAGTTCCACTCGATGTC; CrGPxC-S: CCTAACACCTGTGCTAGCTTC; CrGPxC-A: GCTCTTCAGGTACTTGAACAC; PpGPxA-S: CTTCAGCATGTGGATTGA; PpGPxA.1-A: CTGGATGTCGTTCTCTATCTTTG; PpGPxA.2- A: CTGGGAGAGGAAGACTACCT; PpGPxB-S: GTGGACATTGACGGAGTG; PpGPxB-A: CACTTCAATCTTGGCAAAGAG; SmGPxA.1-S: GCATAGCTTTAGCCTTGTGAC; SmGPxA.2-S: CTTTAGGGCATTTGTTGATT; SmGPxA-A: GTCAATCCACATTGAGAAGC; SmGPxB.1-S: CAGACACAAGAATCCACAGC; SmGPxB.2-S: GCTTGTTCTTCCAGACAC and SmGPxB-A: GTGAAGCCACATTGCGATG genomic and cDNA fragments were amplified to the saturating phase (40 cycles) using standard protocols [95]. The products were separated on 1.2% TAE agarose gels next to size standards (1kb-ladder; Fermentas, St.-Leon-Rot, Germany), stained with ethidium bromide and documented electronically under UV-light [95].

## List of Abbreviations

2CP: 2-Cys peroxiredoxin; 3D: 3-dimensional; aa: amino acid; APx: ascorbate peroxidase, At: *Arabidopsis thaliana*; cDNA: copy DNA; Cr: *Chlamydomonas reinhardtii*; EST: expressed sequence tag; gDNA: genomic DNA; GPx: glutathione peroxidase; hnRNA: heteronuclear RNA; ID. identification number, LHCP: light harvesting complex protein; ORF: open reading frame; Pp: *Physcomitrella patens*; Prx: peroxiredoxin; PrxII: type-II peroxiredoxin; PrxQ: peroxiredoxin Q; RbcS: ribulose-1,5-bisphosphate carboxylase oxygenase small subunit; sAPx: stromal ascorbate peroxidase; ROS: reactive oxygen species; Sm: *Selaginella moellendorffii*; tAPx: thylakoid ascorbate peroxidase

## Authors' contributions

NTP performed the data mining, most of the analysis and was involved in preparation of the manuscript. BW performed the RT-PCR analysis. MB supported data analysis and prepared the manuscript. All authors read and approved the final manuscript.

## Supplementary Material

Additional file 1 **Maximum parsimony tree for APx**. The proteins depicted in Fig. 1A are marked in red. They are compared to all putative fulllength organellar APx listed in PeroxiBase and a selection of extraorganellar APx. PeroxiBasedata (not listed in fig. 1A) are labeled with the PeroxiBase data base IDs. Click here for file

Additional file 2 **Minimum evolution tree for APx**. The proteins depicted in Fig. 1A are marked in red. They are compared to all putative fulllength organellar APx listed in PeroxiBase and a selection of extraorganellar APx. PeroxiBasedata (not listed in fig. 1A) are labeled with the PeroxiBase data base IDs. Click here for file

Additional file 3 **Minimum evolution tree for 2CP**. Phylogramme of the 2CP sequences shown in Fig. 5A (red) and additional 2CP from chlorobionts and cyanobacteria as listed in PeroxiBase [96]. PeroxiBase-data (not listed in fig. 5A) are labeled with the PeroxiBase data base IDs. Click here for file

Additional file 4 **Maximum parsimony tree for 2CP**. Phylogramme of the 2CP sequences shown in Fig. 5A (red) and additional 2CP from chlorobionts and cyanobacteria as listed in PeroxiBase [96]. PeroxiBasedata (not listed in fig. 5A) are labeled with the PeroxiBase data base IDs. Click here for file

Additional file 5 **Maximum parsimony tree for PrxQ**. Phylogramme of the PrxQ sequences shown in Fig. 8A (red) and putative full-length PrxQ sequences of chlorobiont and cyanobacterial origin as listed in PeroxiBase [96]. For PeroxiBase-data the data base IDs are presented in the labels. Click here for file

Additional file 6 **Minimum evolution tree for PrxQ**. Phylogramme of the PrxQ sequences shown in Fig. 8A (red) and putative full-length PrxQ sequences of chlorobiont and cyanobacterial origin as listed in PeroxiBase [96]. For PeroxiBase-data the data base IDs are presented in the labels. Click here for file

Additional file 7 **Maximum parsimony tree for PrxII**. Phylogramme of the PrxII sequences shown in Fig. 11A (red) and a selection of PrxII full length sequences listed in PeroxiBase [96]. PeroxiBase-data (not listed in fig. 11A) are labeled with the PeroxiBase data base IDs. Click here for file

Additional file 8 **Minimum evolution tree for PrxII**. Phylogramme of the PrxII sequences shown in Fig. 11A (red) and a selection of PrxII full length sequences listed in PeroxiBase [96]. PeroxiBase-data (not listed in fig. 11A) are labeled with the PeroxiBase data base IDs. Click here for file

Additional file 9 **Maximum parsimony tree for GPx**. Phylogram of the GPx sequences shown in Fig. 14A (red) and a selection of plant GPx full length sequences listed in PeroxiBase [96]. PeroxiBase-data (not listed in fig. 14A) are labeled with the PeroxiBase data base IDs. Click here for file

Additional file 10 **Minimum evolution tree for GPx**. Phylogram of the GPx sequences shown in Fig. 14A (red) and a selection of plant GPx full length sequences listed in PeroxiBase [96]. PeroxiBase-data (not listed in fig. 14A) are labeled with the PeroxiBase data base IDs. Click here for file
