# Supplementary material for: Comparison of the chloroplast peroxidase system in the chlorophyte Chlamydomonas reinhardtii, the bryophyte Physcomitrella patens, the lycophyte Selaginella moellendorffii and the seed plant Arabidopsis thaliana
Source: BMC Plant Biol. 2010 Jun 28;10:133. doi: 10.1186/1471-2229-10-133 (PMC3095285; doi:10.1186/1471-2229-10-133)
Supplement: Additional file 1 — Maximum parsimony tree for APx. The proteins depicted in Fig. 1A are marked in red. They are compared to all putative fulllength organellar APx listed in PeroxiBase and a selection of extraorganellar APx. PeroxiBasedata (not listed in fig. 1A) are labeled with the PeroxiBase data base IDs. [file 1471-2229-10-133-S1.PPT]

## Slide 1
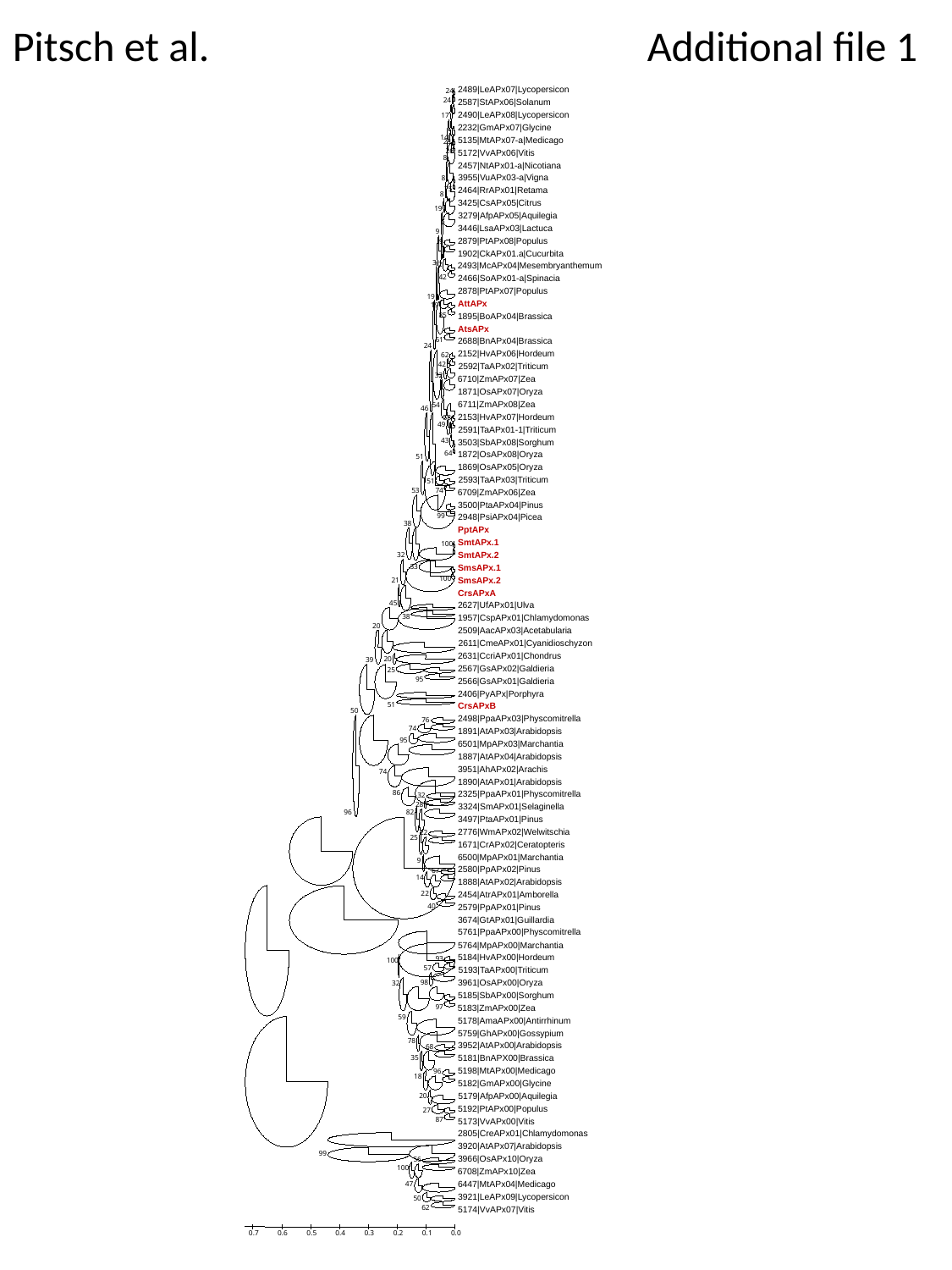

# Pitsch et al.				Additional file 1
 2489|LeAPx07|Lycopersicon
24
24
 2587|StAPx06|Solanum
 2490|LeAPx08|Lycopersicon
17
 2232|GmAPx07|Glycine
14
 5135|MtAPx07-a|Medicago
23
26
 5172|VvAPx06|Vitis
8
 2457|NtAPx01-a|Nicotiana
 3955|VuAPx03-a|Vigna
8
75
 2464|RrAPx01|Retama
8
 3425|CsAPx05|Citrus
19
 3279|AfpAPx05|Aquilegia
 3446|LsaAPx03|Lactuca
9
 2879|PtAPx08|Populus
9
 1902|CkAPx01.a|Cucurbita
3
12
 2493|McAPx04|Mesembryanthemum
42
 2466|SoAPx01-a|Spinacia
 2878|PtAPx07|Populus
19
 AttAPx
19
85
 1895|BoAPx04|Brassica
 AtsAPx
61
 2688|BnAPx04|Brassica
24
 2152|HvAPx06|Hordeum
62
42
 2592|TaAPx02|Triticum
32
 6710|ZmAPx07|Zea
 1871|OsAPx07|Oryza
 6711|ZmAPx08|Zea
54
46
 2153|HvAPx07|Hordeum
58
49
 2591|TaAPx01-1|Triticum
43
 3503|SbAPx08|Sorghum
64
 1872|OsAPx08|Oryza
51
 1869|OsAPx05|Oryza
 2593|TaAPx03|Triticum
51
53
74
 6709|ZmAPx06|Zea
 3500|PtaAPx04|Pinus
99
 2948|PsiAPx04|Picea
38
 PptAPx
 SmtAPx.1
100
 SmtAPx.2
32
33
 SmsAPx.1
100
 SmsAPx.2
21
 CrsAPxA
45
 2627|UfAPx01|Ulva
38
 1957|CspAPx01|Chlamydomonas
20
 2509|AacAPx03|Acetabularia
 2611|CmeAPx01|Cyanidioschyzon
 2631|CcriAPx01|Chondrus
20
39
 2567|GsAPx02|Galdieria
25
95
 2566|GsAPx01|Galdieria
 2406|PyAPx|Porphyra
51
 CrsAPxB
50
 2498|PpaAPx03|Physcomitrella
76
74
 1891|AtAPx03|Arabidopsis
95
 6501|MpAPx03|Marchantia
 1887|AtAPx04|Arabidopsis
 3951|AhAPx02|Arachis
74
 1890|AtAPx01|Arabidopsis
86
 2325|PpaAPx01|Physcomitrella
32
28
 3324|SmAPx01|Selaginella
82
96
 3497|PtaAPx01|Pinus
 2776|WmAPx02|Welwitschia
22
25
 1671|CrAPx02|Ceratopteris
 6500|MpAPx01|Marchantia
9
 2580|PpAPx02|Pinus
67
14
 1888|AtAPx02|Arabidopsis
22
 2454|AtrAPx01|Amborella
40
 2579|PpAPx01|Pinus
 3674|GtAPx01|Guillardia
 5761|PpaAPx00|Physcomitrella
 5764|MpAPx00|Marchantia
 5184|HvAPx00|Hordeum
93
100
57
 5193|TaAPx00|Triticum
 3961|OsAPx00|Oryza
98
32
 5185|SbAPx00|Sorghum
97
 5183|ZmAPx00|Zea
59
 5178|AmaAPx00|Antirrhinum
 5759|GhAPx00|Gossypium
78
 3952|AtAPx00|Arabidopsis
68
 5181|BnAPX00|Brassica
35
 5198|MtAPx00|Medicago
96
18
 5182|GmAPx00|Glycine
 5179|AfpAPx00|Aquilegia
20
 5192|PtAPx00|Populus
27
87
 5173|VvAPx00|Vitis
 2805|CreAPx01|Chlamydomonas
 3920|AtAPx07|Arabidopsis
99
 3966|OsAPx10|Oryza
56
100
 6708|ZmAPx10|Zea
 6447|MtAPx04|Medicago
47
 3921|LeAPx09|Lycopersicon
50
62
 5174|VvAPx07|Vitis
0.7
0.6
0.5
0.4
0.3
0.2
0.1
0.0
