# Supplementary material for: Comparison of the chloroplast peroxidase system in the chlorophyte Chlamydomonas reinhardtii, the bryophyte Physcomitrella patens, the lycophyte Selaginella moellendorffii and the seed plant Arabidopsis thaliana
Source: BMC Plant Biol. 2010 Jun 28;10:133. doi: 10.1186/1471-2229-10-133 (PMC3095285; doi:10.1186/1471-2229-10-133)
Supplement: Additional file 2 — Minimum evolution tree for APx. The proteins depicted in Fig. 1A are marked in red. They are compared to all putative fulllength organellar APx listed in PeroxiBase and a selection of extraorganellar APx. PeroxiBasedata (not listed in fig. 1A) are labeled with the PeroxiBase data base IDs. [file 1471-2229-10-133-S2.PPT]

## Slide 1
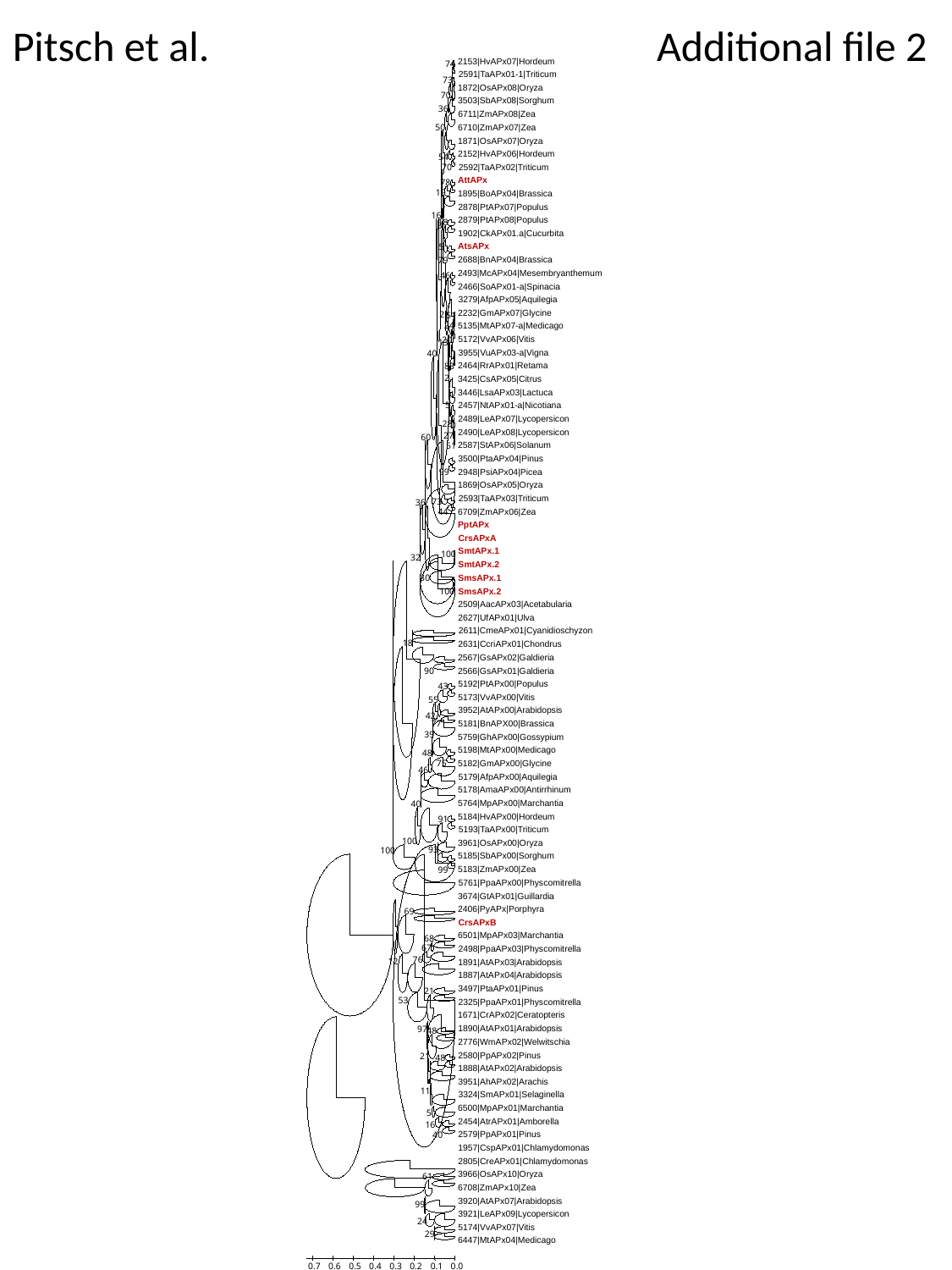

# Pitsch et al.				 Additional file 2
 2153|HvAPx07|Hordeum
 2591|TaAPx01-1|Triticum
 1872|OsAPx08|Oryza
 3503|SbAPx08|Sorghum
 6711|ZmAPx08|Zea
 6710|ZmAPx07|Zea
 1871|OsAPx07|Oryza
 2152|HvAPx06|Hordeum
 2592|TaAPx02|Triticum
 AttAPx
 1895|BoAPx04|Brassica
 2878|PtAPx07|Populus
 2879|PtAPx08|Populus
 1902|CkAPx01.a|Cucurbita
 AtsAPx
 2688|BnAPx04|Brassica
 2493|McAPx04|Mesembryanthemum
 2466|SoAPx01-a|Spinacia
 3279|AfpAPx05|Aquilegia
 2232|GmAPx07|Glycine
 5135|MtAPx07-a|Medicago
 5172|VvAPx06|Vitis
 3955|VuAPx03-a|Vigna
 2464|RrAPx01|Retama
 3425|CsAPx05|Citrus
 3446|LsaAPx03|Lactuca
 2457|NtAPx01-a|Nicotiana
 2489|LeAPx07|Lycopersicon
 2490|LeAPx08|Lycopersicon
 2587|StAPx06|Solanum
 3500|PtaAPx04|Pinus
 2948|PsiAPx04|Picea
 1869|OsAPx05|Oryza
 2593|TaAPx03|Triticum
 6709|ZmAPx06|Zea
 PptAPx
 CrsAPxA
 SmtAPx.1
 SmtAPx.2
 SmsAPx.1
 SmsAPx.2
 2509|AacAPx03|Acetabularia
 2627|UfAPx01|Ulva
 2611|CmeAPx01|Cyanidioschyzon
 2631|CcriAPx01|Chondrus
 2567|GsAPx02|Galdieria
 2566|GsAPx01|Galdieria
 5192|PtAPx00|Populus
 5173|VvAPx00|Vitis
 3952|AtAPx00|Arabidopsis
 5181|BnAPX00|Brassica
 5759|GhAPx00|Gossypium
 5198|MtAPx00|Medicago
 5182|GmAPx00|Glycine
 5179|AfpAPx00|Aquilegia
 5178|AmaAPx00|Antirrhinum
 5764|MpAPx00|Marchantia
 5184|HvAPx00|Hordeum
 5193|TaAPx00|Triticum
 3961|OsAPx00|Oryza
 5185|SbAPx00|Sorghum
 5183|ZmAPx00|Zea
 5761|PpaAPx00|Physcomitrella
 3674|GtAPx01|Guillardia
 2406|PyAPx|Porphyra
 CrsAPxB
 6501|MpAPx03|Marchantia
 2498|PpaAPx03|Physcomitrella
 1891|AtAPx03|Arabidopsis
74
73
70
36
50
54
70
78
19
16
18
3
5
79
46
2
51
24
20
13
40
83
2
5
28
27
60
51
99
73
36
44
100
32
30
100
18
90
43
55
42
77
39
48
75
46
40
91
100
93
100
99
69
68
67
76
12
 1887|AtAPx04|Arabidopsis
 3497|PtaAPx01|Pinus
21
53
 2325|PpaAPx01|Physcomitrella
 1671|CrAPx02|Ceratopteris
 1890|AtAPx01|Arabidopsis
97
48
 2776|WmAPx02|Welwitschia
 2580|PpAPx02|Pinus
21
48
 1888|AtAPx02|Arabidopsis
 3951|AhAPx02|Arachis
11
 3324|SmAPx01|Selaginella
 6500|MpAPx01|Marchantia
5
 2454|AtrAPx01|Amborella
16
 2579|PpAPx01|Pinus
40
 1957|CspAPx01|Chlamydomonas
 2805|CreAPx01|Chlamydomonas
 3966|OsAPx10|Oryza
61
 6708|ZmAPx10|Zea
 3920|AtAPx07|Arabidopsis
99
 3921|LeAPx09|Lycopersicon
24
 5174|VvAPx07|Vitis
29
 6447|MtAPx04|Medicago
0.7
0.6
0.5
0.4
0.3
0.2
0.1
0.0
