# Supplementary material for: Comparison of the chloroplast peroxidase system in the chlorophyte Chlamydomonas reinhardtii, the bryophyte Physcomitrella patens, the lycophyte Selaginella moellendorffii and the seed plant Arabidopsis thaliana
Source: BMC Plant Biol. 2010 Jun 28;10:133. doi: 10.1186/1471-2229-10-133 (PMC3095285; doi:10.1186/1471-2229-10-133)
Supplement: Additional file 3 — Minimum evolution tree for 2CP. Phylogramme of the 2CP sequences shown in Fig. 5A (red) and additional 2CP from chlorobionts and cyanobacteria as listed in PeroxiBase [96]. PeroxiBase-data (not listed in fig. 5A) are labeled with the PeroxiBase data base IDs. [file 1471-2229-10-133-S3.PPT]

## Slide 1
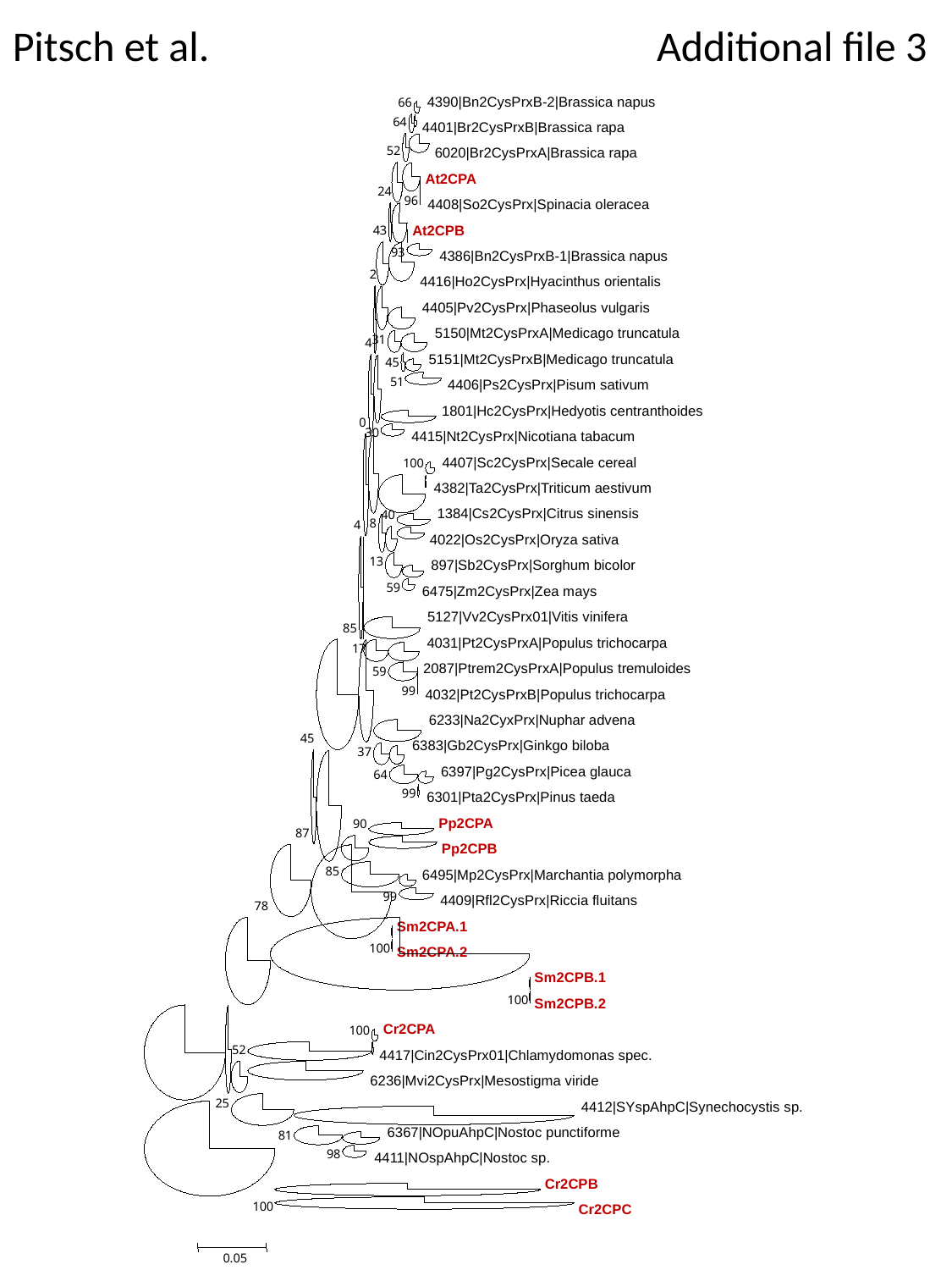

# Pitsch et al.				 Additional file 3
 4390|Bn2CysPrxB-2|Brassica napus
66
64
 4401|Br2CysPrxB|Brassica rapa
52
 6020|Br2CysPrxA|Brassica rapa
 At2CPA
24
96
 4408|So2CysPrx|Spinacia oleracea
 At2CPB
43
93
 4386|Bn2CysPrxB-1|Brassica napus
2
 4416|Ho2CysPrx|Hyacinthus orientalis
 4405|Pv2CysPrx|Phaseolus vulgaris
 5150|Mt2CysPrxA|Medicago truncatula
31
4
 5151|Mt2CysPrxB|Medicago truncatula
45
51
 4406|Ps2CysPrx|Pisum sativum
 1801|Hc2CysPrx|Hedyotis centranthoides
0
30
 4415|Nt2CysPrx|Nicotiana tabacum
 4407|Sc2CysPrx|Secale cereal
100
 4382|Ta2CysPrx|Triticum aestivum
 1384|Cs2CysPrx|Citrus sinensis
40
8
4
 4022|Os2CysPrx|Oryza sativa
13
 897|Sb2CysPrx|Sorghum bicolor
59
 6475|Zm2CysPrx|Zea mays
 5127|Vv2CysPrx01|Vitis vinifera
85
 4031|Pt2CysPrxA|Populus trichocarpa
17
 2087|Ptrem2CysPrxA|Populus tremuloides
59
99
 4032|Pt2CysPrxB|Populus trichocarpa
 6233|Na2CyxPrx|Nuphar advena
45
 6383|Gb2CysPrx|Ginkgo biloba
37
 6397|Pg2CysPrx|Picea glauca
64
99
 6301|Pta2CysPrx|Pinus taeda
 Pp2CPA
90
87
 Pp2CPB
85
 6495|Mp2CysPrx|Marchantia polymorpha
99
 4409|Rfl2CysPrx|Riccia fluitans
78
 Sm2CPA.1
100
 Sm2CPA.2
 Sm2CPB.1
100
 Sm2CPB.2
 Cr2CPA
100
52
 4417|Cin2CysPrx01|Chlamydomonas spec.
 6236|Mvi2CysPrx|Mesostigma viride
25
 4412|SYspAhpC|Synechocystis sp.
 6367|NOpuAhpC|Nostoc punctiforme
81
98
 4411|NOspAhpC|Nostoc sp.
 Cr2CPB
100
 Cr2CPC
0.05
