# Supplementary material for: Comparison of the chloroplast peroxidase system in the chlorophyte Chlamydomonas reinhardtii, the bryophyte Physcomitrella patens, the lycophyte Selaginella moellendorffii and the seed plant Arabidopsis thaliana
Source: BMC Plant Biol. 2010 Jun 28;10:133. doi: 10.1186/1471-2229-10-133 (PMC3095285; doi:10.1186/1471-2229-10-133)
Supplement: Additional file 4 — Maximum parsimony tree for 2CP. Phylogramme of the 2CP sequences shown in Fig. 5A (red) and additional 2CP from chlorobionts and cyanobacteria as listed in PeroxiBase [96]. PeroxiBasedata (not listed in fig. 5A) are labeled with the PeroxiBase data base IDs. [file 1471-2229-10-133-S4.PPT]

## Slide 1
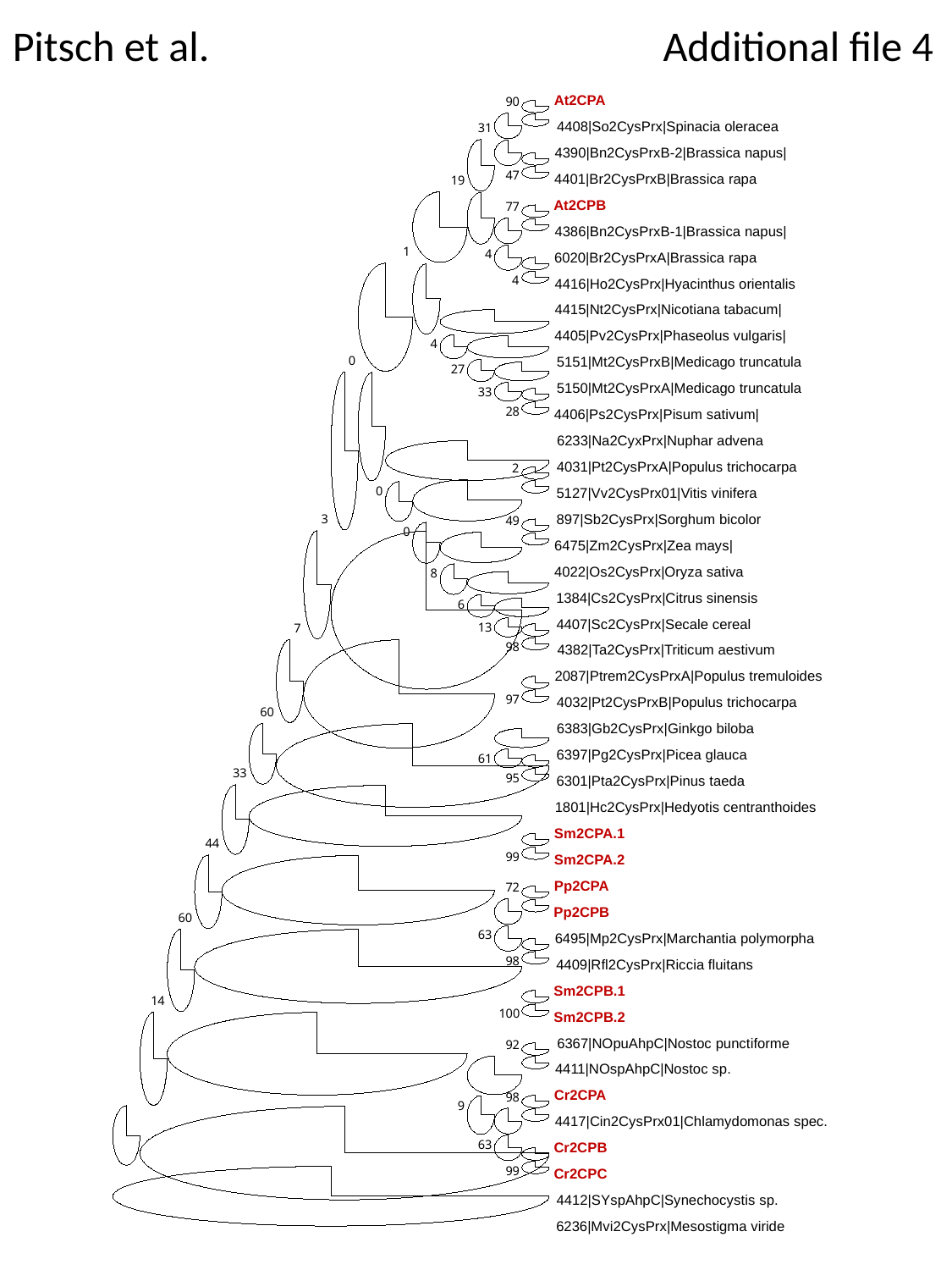

# Pitsch et al.			 Additional file 4
 At2CPA
90
 4408|So2CysPrx|Spinacia oleracea
31
 4390|Bn2CysPrxB-2|Brassica napus|
47
 4401|Br2CysPrxB|Brassica rapa
19
 At2CPB
77
 4386|Bn2CysPrxB-1|Brassica napus|
1
4
 6020|Br2CysPrxA|Brassica rapa
4
 4416|Ho2CysPrx|Hyacinthus orientalis
 4415|Nt2CysPrx|Nicotiana tabacum|
 4405|Pv2CysPrx|Phaseolus vulgaris|
4
0
 5151|Mt2CysPrxB|Medicago truncatula
27
 5150|Mt2CysPrxA|Medicago truncatula
33
28
 4406|Ps2CysPrx|Pisum sativum|
 6233|Na2CyxPrx|Nuphar advena
 4031|Pt2CysPrxA|Populus trichocarpa
2
0
 5127|Vv2CysPrx01|Vitis vinifera
 897|Sb2CysPrx|Sorghum bicolor
3
49
0
 6475|Zm2CysPrx|Zea mays|
 4022|Os2CysPrx|Oryza sativa
8
 1384|Cs2CysPrx|Citrus sinensis
6
 4407|Sc2CysPrx|Secale cereal
13
7
98
 4382|Ta2CysPrx|Triticum aestivum
 2087|Ptrem2CysPrxA|Populus tremuloides
97
 4032|Pt2CysPrxB|Populus trichocarpa
60
 6383|Gb2CysPrx|Ginkgo biloba
 6397|Pg2CysPrx|Picea glauca
61
33
95
 6301|Pta2CysPrx|Pinus taeda
 1801|Hc2CysPrx|Hedyotis centranthoides
 Sm2CPA.1
44
99
 Sm2CPA.2
 Pp2CPA
72
 Pp2CPB
60
63
 6495|Mp2CysPrx|Marchantia polymorpha
98
 4409|Rfl2CysPrx|Riccia fluitans
 Sm2CPB.1
14
100
 Sm2CPB.2
 6367|NOpuAhpC|Nostoc punctiforme
92
 4411|NOspAhpC|Nostoc sp.
 Cr2CPA
98
9
 4417|Cin2CysPrx01|Chlamydomonas spec.
63
 Cr2CPB
99
 Cr2CPC
 4412|SYspAhpC|Synechocystis sp.
 6236|Mvi2CysPrx|Mesostigma viride
