# Supplementary material for: Comparison of the chloroplast peroxidase system in the chlorophyte Chlamydomonas reinhardtii, the bryophyte Physcomitrella patens, the lycophyte Selaginella moellendorffii and the seed plant Arabidopsis thaliana
Source: BMC Plant Biol. 2010 Jun 28;10:133. doi: 10.1186/1471-2229-10-133 (PMC3095285; doi:10.1186/1471-2229-10-133)
Supplement: Additional file 5 — Maximum parsimony tree for PrxQ. Phylogramme of the PrxQ sequences shown in Fig. 8A (red) and putative full-length PrxQ sequences of chlorobiont and cyanobacterial origin as listed in PeroxiBase [96]. For PeroxiBase-data the data base IDs are presented in the labels. [file 1471-2229-10-133-S5.PPT]

## Slide 1
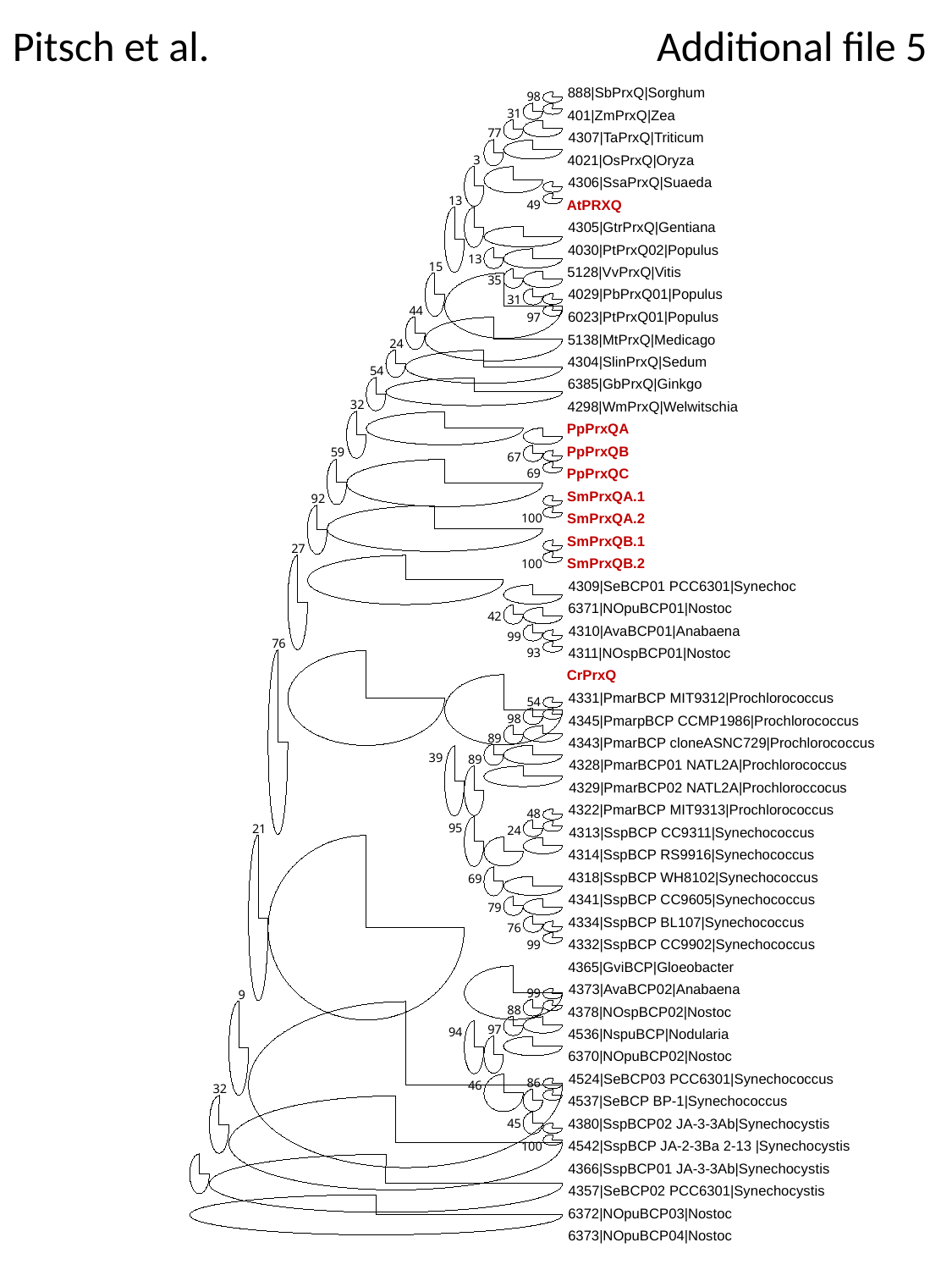

# Pitsch et al.				 Additional file 5
 888|SbPrxQ|Sorghum
98
31
 401|ZmPrxQ|Zea
77
 4307|TaPrxQ|Triticum
 4021|OsPrxQ|Oryza
3
 4306|SsaPrxQ|Suaeda
13
 AtPRXQ
49
 4305|GtrPrxQ|Gentiana
 4030|PtPrxQ02|Populus
13
15
 5128|VvPrxQ|Vitis
35
 4029|PbPrxQ01|Populus
31
44
 6023|PtPrxQ01|Populus
97
 5138|MtPrxQ|Medicago
24
 4304|SlinPrxQ|Sedum
54
 6385|GbPrxQ|Ginkgo
32
 4298|WmPrxQ|Welwitschia
 PpPrxQA
 PpPrxQB
59
67
 PpPrxQC
69
 SmPrxQA.1
92
 SmPrxQA.2
100
 SmPrxQB.1
27
 SmPrxQB.2
100
 4309|SeBCP01 PCC6301|Synechoc
 6371|NOpuBCP01|Nostoc
42
 4310|AvaBCP01|Anabaena
99
76
 4311|NOspBCP01|Nostoc
93
 CrPrxQ
 4331|PmarBCP MIT9312|Prochlorococcus
54
98
 4345|PmarpBCP CCMP1986|Prochlorococcus
89
 4343|PmarBCP cloneASNC729|Prochlorococcus
39
89
 4328|PmarBCP01 NATL2A|Prochlorococcus
 4329|PmarBCP02 NATL2A|Prochloroccocus
 4322|PmarBCP MIT9313|Prochlorococcus
48
95
24
 4313|SspBCP CC9311|Synechococcus
 4314|SspBCP RS9916|Synechococcus
 4318|SspBCP WH8102|Synechococcus
69
 4341|SspBCP CC9605|Synechococcus
79
 4334|SspBCP BL107|Synechococcus
76
 4332|SspBCP CC9902|Synechococcus
99
 4365|GviBCP|Gloeobacter
 4373|AvaBCP02|Anabaena
99
88
 4378|NOspBCP02|Nostoc
97
94
 4536|NspuBCP|Nodularia
 6370|NOpuBCP02|Nostoc
 4524|SeBCP03 PCC6301|Synechococcus
86
46
 4537|SeBCP BP-1|Synechococcus
 4380|SspBCP02 JA-3-3Ab|Synechocystis
45
 4542|SspBCP JA-2-3Ba 2-13 |Synechocystis
100
 4366|SspBCP01 JA-3-3Ab|Synechocystis
 4357|SeBCP02 PCC6301|Synechocystis
 6372|NOpuBCP03|Nostoc
 6373|NOpuBCP04|Nostoc
21
9
32
