# Supplementary material for: Comparison of the chloroplast peroxidase system in the chlorophyte Chlamydomonas reinhardtii, the bryophyte Physcomitrella patens, the lycophyte Selaginella moellendorffii and the seed plant Arabidopsis thaliana
Source: BMC Plant Biol. 2010 Jun 28;10:133. doi: 10.1186/1471-2229-10-133 (PMC3095285; doi:10.1186/1471-2229-10-133)
Supplement: Additional file 7 — Maximum parsimony tree for PrxII. Phylogramme of the PrxII sequences shown in Fig. 11A (red) and a selection of PrxII full length sequences listed in PeroxiBase [96]. PeroxiBase-data (not listed in fig. 11A) are labeled with the PeroxiBase data base IDs. [file 1471-2229-10-133-S7.PPT]

## Slide 1
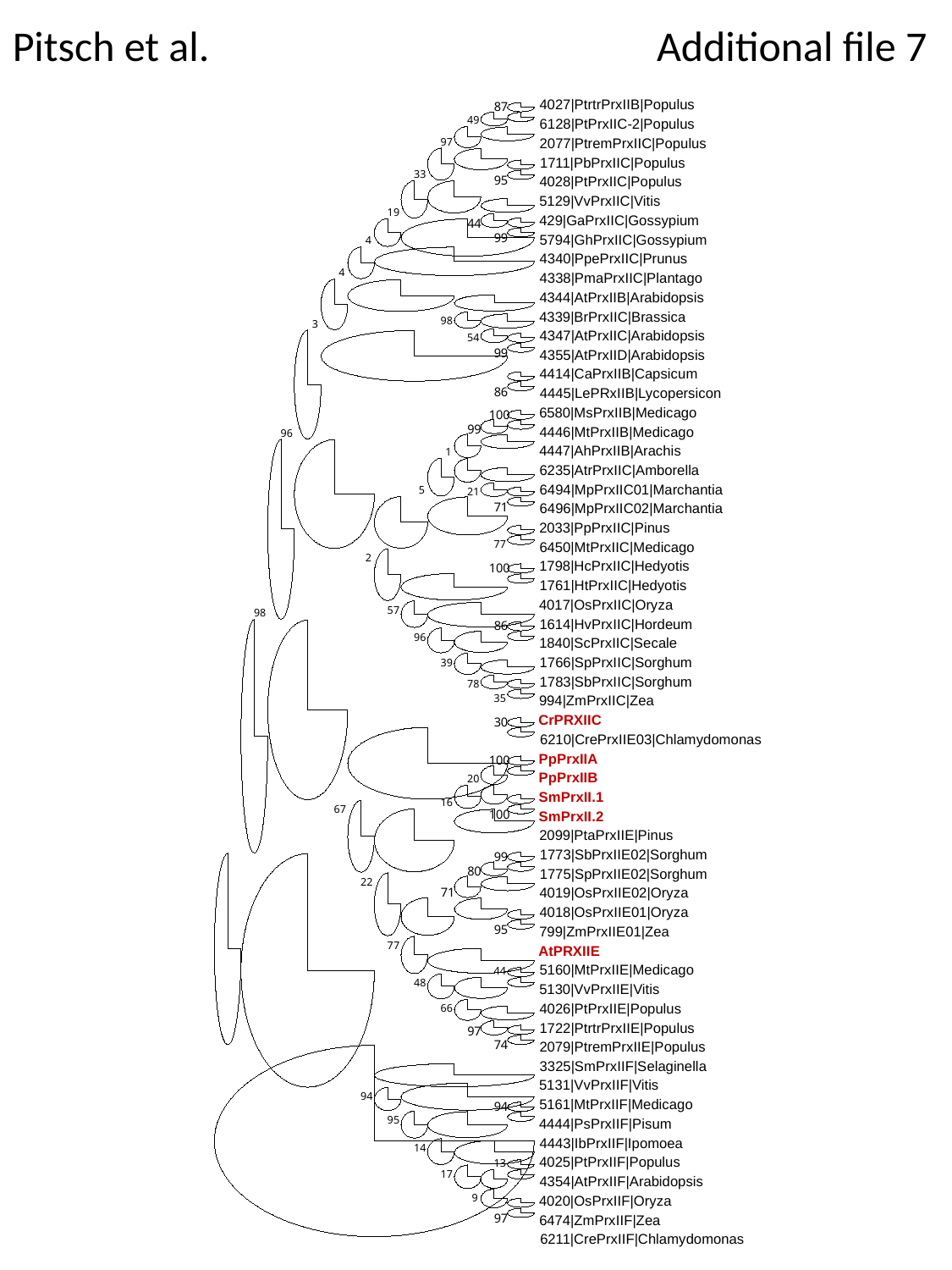

# Pitsch et al.				 Additional file 7
 4027|PtrtrPrxIIB|Populus
87
49
 6128|PtPrxIIC-2|Populus
 2077|PtremPrxIIC|Populus
97
 1711|PbPrxIIC|Populus
33
95
 4028|PtPrxIIC|Populus
 5129|VvPrxIIC|Vitis
19
 429|GaPrxIIC|Gossypium
44
99
 5794|GhPrxIIC|Gossypium
4
 4340|PpePrxIIC|Prunus
4
 4338|PmaPrxIIC|Plantago
 4344|AtPrxIIB|Arabidopsis
 4339|BrPrxIIC|Brassica
98
3
 4347|AtPrxIIC|Arabidopsis
54
99
 4355|AtPrxIID|Arabidopsis
 4414|CaPrxIIB|Capsicum
86
 4445|LePRxIIB|Lycopersicon
 6580|MsPrxIIB|Medicago
100
99
 4446|MtPrxIIB|Medicago
96
 4447|AhPrxIIB|Arachis
1
 6235|AtrPrxIIC|Amborella
 6494|MpPrxIIC01|Marchantia
5
21
71
 6496|MpPrxIIC02|Marchantia
 2033|PpPrxIIC|Pinus
77
 6450|MtPrxIIC|Medicago
2
 1798|HcPrxIIC|Hedyotis
100
 1761|HtPrxIIC|Hedyotis
 4017|OsPrxIIC|Oryza
57
98
 1614|HvPrxIIC|Hordeum
86
96
 1840|ScPrxIIC|Secale
 1766|SpPrxIIC|Sorghum
39
 1783|SbPrxIIC|Sorghum
78
35
 994|ZmPrxIIC|Zea
 CrPRXIIC
30
 6210|CrePrxIIE03|Chlamydomonas
 PpPrxIIA
100
 PpPrxIIB
20
 SmPrxII.1
16
67
100
 SmPrxII.2
 2099|PtaPrxIIE|Pinus
 1773|SbPrxIIE02|Sorghum
99
80
 1775|SpPrxIIE02|Sorghum
22
 4019|OsPrxIIE02|Oryza
71
 4018|OsPrxIIE01|Oryza
95
 799|ZmPrxIIE01|Zea
77
 AtPRXIIE
 5160|MtPrxIIE|Medicago
44
48
 5130|VvPrxIIE|Vitis
 4026|PtPrxIIE|Populus
66
 1722|PtrtrPrxIIE|Populus
97
74
 2079|PtremPrxIIE|Populus
 3325|SmPrxIIF|Selaginella
 5131|VvPrxIIF|Vitis
94
 5161|MtPrxIIF|Medicago
94
95
 4444|PsPrxIIF|Pisum
 4443|IbPrxIIF|Ipomoea
14
 4025|PtPrxIIF|Populus
13
17
 4354|AtPrxIIF|Arabidopsis
9
 4020|OsPrxIIF|Oryza
97
 6474|ZmPrxIIF|Zea
 6211|CrePrxIIF|Chlamydomonas
