# Supplementary material for: Comparison of the chloroplast peroxidase system in the chlorophyte Chlamydomonas reinhardtii, the bryophyte Physcomitrella patens, the lycophyte Selaginella moellendorffii and the seed plant Arabidopsis thaliana
Source: BMC Plant Biol. 2010 Jun 28;10:133. doi: 10.1186/1471-2229-10-133 (PMC3095285; doi:10.1186/1471-2229-10-133)
Supplement: Additional file 8 — Minimum evolution tree for PrxII. Phylogramme of the PrxII sequences shown in Fig. 11A (red) and a selection of PrxII full length sequences listed in PeroxiBase [96]. PeroxiBase-data (not listed in fig. 11A) are labeled with the PeroxiBase data base IDs. [file 1471-2229-10-133-S8.PPT]

## Slide 1
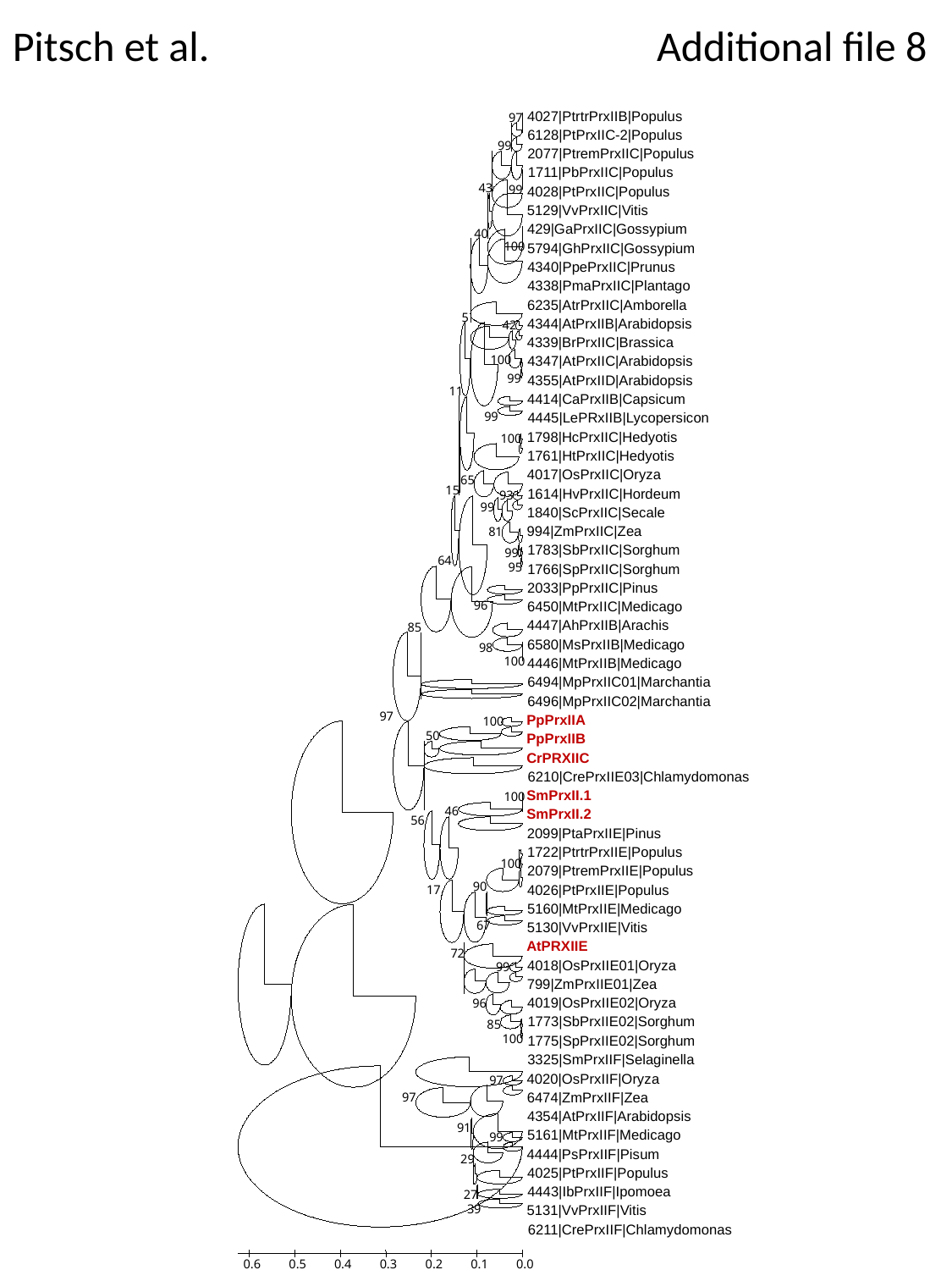

# Pitsch et al.				 Additional file 8
 4027|PtrtrPrxIIB|Populus
 6128|PtPrxIIC-2|Populus
 2077|PtremPrxIIC|Populus
 1711|PbPrxIIC|Populus
 4028|PtPrxIIC|Populus
 5129|VvPrxIIC|Vitis
 429|GaPrxIIC|Gossypium
 5794|GhPrxIIC|Gossypium
 4340|PpePrxIIC|Prunus
 4338|PmaPrxIIC|Plantago
 6235|AtrPrxIIC|Amborella
 4344|AtPrxIIB|Arabidopsis
 4339|BrPrxIIC|Brassica
 4347|AtPrxIIC|Arabidopsis
 4355|AtPrxIID|Arabidopsis
 4414|CaPrxIIB|Capsicum
 4445|LePRxIIB|Lycopersicon
 1798|HcPrxIIC|Hedyotis
 1761|HtPrxIIC|Hedyotis
 4017|OsPrxIIC|Oryza
 1614|HvPrxIIC|Hordeum
 1840|ScPrxIIC|Secale
 994|ZmPrxIIC|Zea
 1783|SbPrxIIC|Sorghum
 1766|SpPrxIIC|Sorghum
 2033|PpPrxIIC|Pinus
 6450|MtPrxIIC|Medicago
 4447|AhPrxIIB|Arachis
 6580|MsPrxIIB|Medicago
100
 4446|MtPrxIIB|Medicago
 6494|MpPrxIIC01|Marchantia
 6496|MpPrxIIC02|Marchantia
97
 PpPrxIIA
100
50
 PpPrxIIB
 CrPRXIIC
 6210|CrePrxIIE03|Chlamydomonas
 SmPrxII.1
100
46
 SmPrxII.2
56
 2099|PtaPrxIIE|Pinus
 1722|PtrtrPrxIIE|Populus
100
 2079|PtremPrxIIE|Populus
90
 4026|PtPrxIIE|Populus
17
 5160|MtPrxIIE|Medicago
67
 5130|VvPrxIIE|Vitis
 AtPRXIIE
72
 4018|OsPrxIIE01|Oryza
99
 799|ZmPrxIIE01|Zea
 4019|OsPrxIIE02|Oryza
96
 1773|SbPrxIIE02|Sorghum
85
100
 1775|SpPrxIIE02|Sorghum
 3325|SmPrxIIF|Selaginella
 4020|OsPrxIIF|Oryza
97
 6474|ZmPrxIIF|Zea
97
 4354|AtPrxIIF|Arabidopsis
91
 5161|MtPrxIIF|Medicago
99
 4444|PsPrxIIF|Pisum
29
 4025|PtPrxIIF|Populus
 4443|IbPrxIIF|Ipomoea
27
39
 5131|VvPrxIIF|Vitis
 6211|CrePrxIIF|Chlamydomonas
97
99
43
99
40
100
5
42
100
99
11
99
100
65
15
93
99
81
99
64
95
96
85
98
0.6
0.5
0.4
0.3
0.2
0.1
0.0
