# Supplementary material for: Comparison of the chloroplast peroxidase system in the chlorophyte Chlamydomonas reinhardtii, the bryophyte Physcomitrella patens, the lycophyte Selaginella moellendorffii and the seed plant Arabidopsis thaliana
Source: BMC Plant Biol. 2010 Jun 28;10:133. doi: 10.1186/1471-2229-10-133 (PMC3095285; doi:10.1186/1471-2229-10-133)
Supplement: Additional file 9 — Maximum parsimony tree for GPx. Phylogram of the GPx sequences shown in Fig. 14A (red) and a selection of plant GPx full length sequences listed in PeroxiBase [96]. PeroxiBase-data (not listed in fig. 14A) are labeled with the PeroxiBase data base IDs. [file 1471-2229-10-133-S9.PPT]

## Slide 1
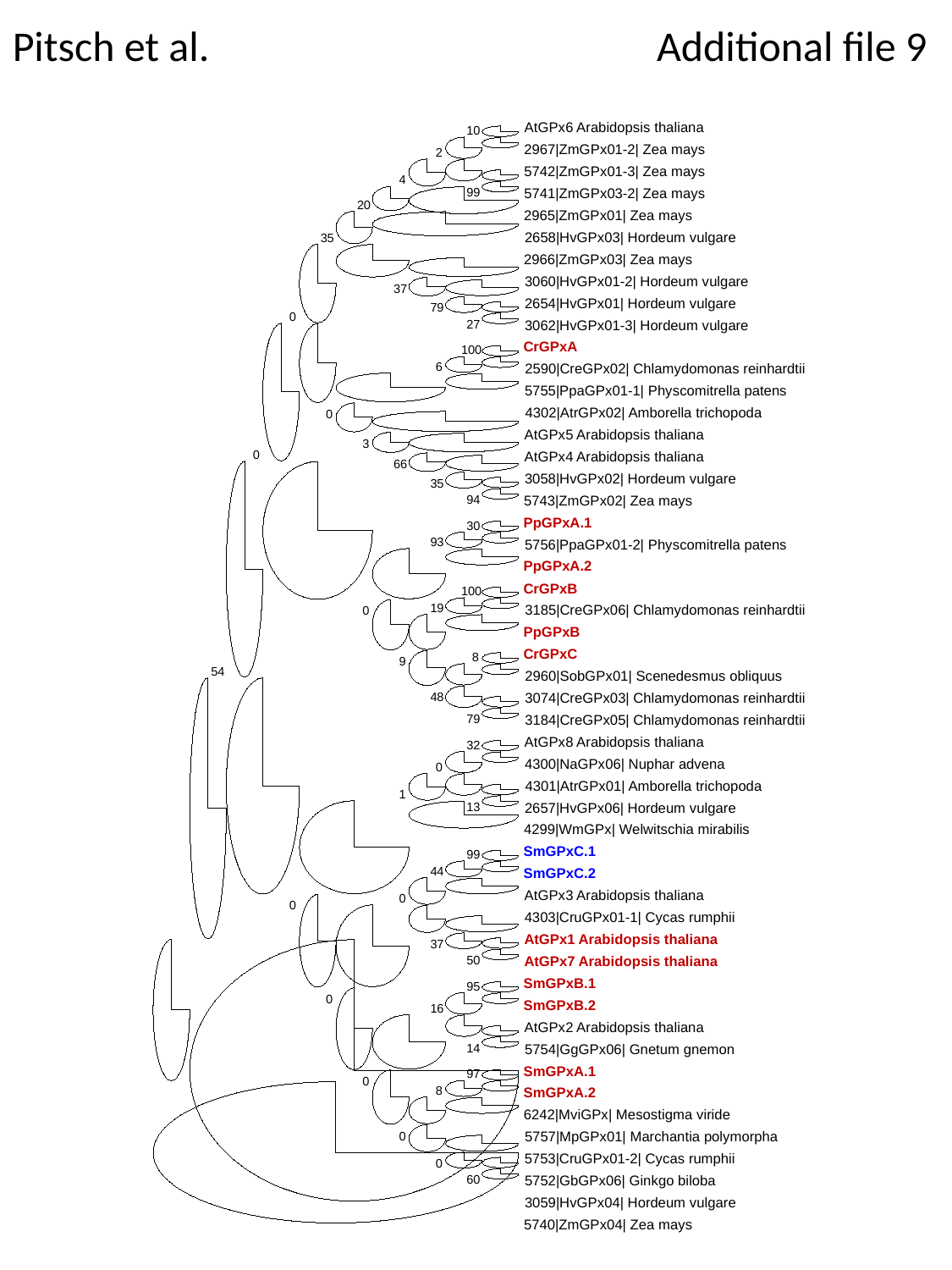

# Pitsch et al.				 Additional file 9
 AtGPx6 Arabidopsis thaliana
10
 2967|ZmGPx01-2| Zea mays
2
 5742|ZmGPx01-3| Zea mays
4
 5741|ZmGPx03-2| Zea mays
99
20
 2965|ZmGPx01| Zea mays
 2658|HvGPx03| Hordeum vulgare
35
 2966|ZmGPx03| Zea mays
 3060|HvGPx01-2| Hordeum vulgare
37
 2654|HvGPx01| Hordeum vulgare
79
0
 3062|HvGPx01-3| Hordeum vulgare
27
 CrGPxA
100
6
 2590|CreGPx02| Chlamydomonas reinhardtii
 5755|PpaGPx01-1| Physcomitrella patens
 4302|AtrGPx02| Amborella trichopoda
0
 AtGPx5 Arabidopsis thaliana
3
0
 AtGPx4 Arabidopsis thaliana
66
 3058|HvGPx02| Hordeum vulgare
35
 5743|ZmGPx02| Zea mays
94
 PpGPxA.1
30
93
 5756|PpaGPx01-2| Physcomitrella patens
 PpGPxA.2
 CrGPxB
100
19
 3185|CreGPx06| Chlamydomonas reinhardtii
0
 PpGPxB
 CrGPxC
8
9
54
 2960|SobGPx01| Scenedesmus obliquus
 3074|CreGPx03| Chlamydomonas reinhardtii
48
 3184|CreGPx05| Chlamydomonas reinhardtii
79
 AtGPx8 Arabidopsis thaliana
32
 4300|NaGPx06| Nuphar advena
0
 4301|AtrGPx01| Amborella trichopoda
1
 2657|HvGPx06| Hordeum vulgare
13
 4299|WmGPx| Welwitschia mirabilis
 SmGPxC.1
99
44
 SmGPxC.2
 AtGPx3 Arabidopsis thaliana
0
0
 4303|CruGPx01-1| Cycas rumphii
 AtGPx1 Arabidopsis thaliana
37
 AtGPx7 Arabidopsis thaliana
50
 SmGPxB.1
95
0
 SmGPxB.2
16
 AtGPx2 Arabidopsis thaliana
 5754|GgGPx06| Gnetum gnemon
14
 SmGPxA.1
97
0
8
 SmGPxA.2
 6242|MviGPx| Mesostigma viride
 5757|MpGPx01| Marchantia polymorpha
0
 5753|CruGPx01-2| Cycas rumphii
0
 5752|GbGPx06| Ginkgo biloba
60
 3059|HvGPx04| Hordeum vulgare
 5740|ZmGPx04| Zea mays
