# Supplementary material for: Comparison of the chloroplast peroxidase system in the chlorophyte Chlamydomonas reinhardtii, the bryophyte Physcomitrella patens, the lycophyte Selaginella moellendorffii and the seed plant Arabidopsis thaliana
Source: BMC Plant Biol. 2010 Jun 28;10:133. doi: 10.1186/1471-2229-10-133 (PMC3095285; doi:10.1186/1471-2229-10-133)
Supplement: Additional file 10 — Minimum evolution tree for GPx. Phylogram of the GPx sequences shown in Fig. 14A (red) and a selection of plant GPx full length sequences listed in PeroxiBase [96]. PeroxiBase-data (not listed in fig. 14A) are labeled with the PeroxiBase data base IDs. [file 1471-2229-10-133-S10.PPT]

## Slide 1
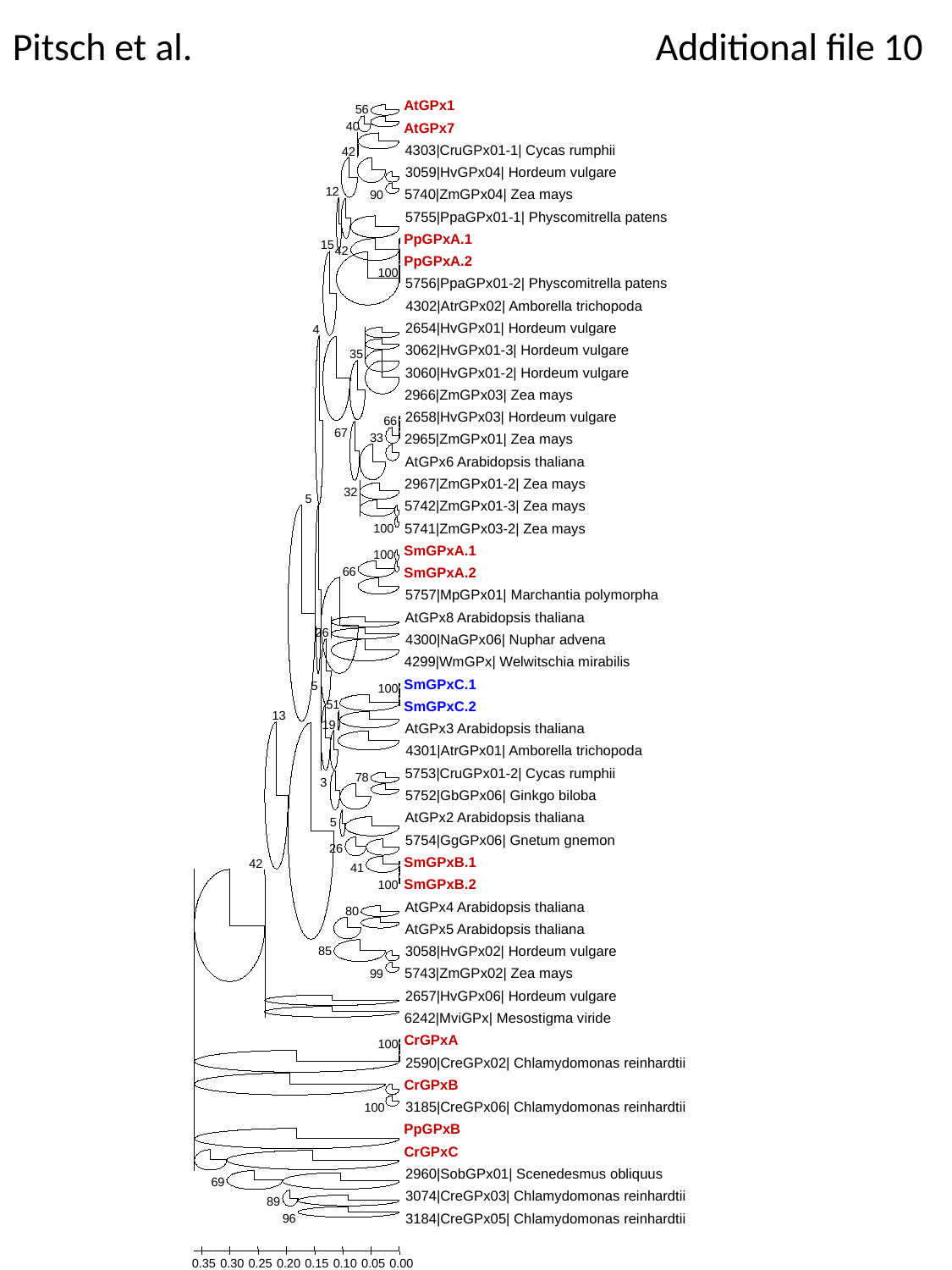

# Pitsch et al.				 Additional file 10
 AtGPx1
56
40
 AtGPx7
 4303|CruGPx01-1| Cycas rumphii
42
 3059|HvGPx04| Hordeum vulgare
12
 5740|ZmGPx04| Zea mays
90
 5755|PpaGPx01-1| Physcomitrella patens
 PpGPxA.1
15
42
 PpGPxA.2
100
 5756|PpaGPx01-2| Physcomitrella patens
 4302|AtrGPx02| Amborella trichopoda
 2654|HvGPx01| Hordeum vulgare
4
 3062|HvGPx01-3| Hordeum vulgare
35
 3060|HvGPx01-2| Hordeum vulgare
 2966|ZmGPx03| Zea mays
 2658|HvGPx03| Hordeum vulgare
66
67
33
 2965|ZmGPx01| Zea mays
 AtGPx6 Arabidopsis thaliana
 2967|ZmGPx01-2| Zea mays
32
5
 5742|ZmGPx01-3| Zea mays
 5741|ZmGPx03-2| Zea mays
100
 SmGPxA.1
100
66
 SmGPxA.2
 5757|MpGPx01| Marchantia polymorpha
 AtGPx8 Arabidopsis thaliana
26
 4300|NaGPx06| Nuphar advena
 4299|WmGPx| Welwitschia mirabilis
 SmGPxC.1
5
100
51
 SmGPxC.2
13
19
 AtGPx3 Arabidopsis thaliana
 4301|AtrGPx01| Amborella trichopoda
 5753|CruGPx01-2| Cycas rumphii
78
3
 5752|GbGPx06| Ginkgo biloba
 AtGPx2 Arabidopsis thaliana
5
 5754|GgGPx06| Gnetum gnemon
26
 SmGPxB.1
42
41
 SmGPxB.2
100
 AtGPx4 Arabidopsis thaliana
80
 AtGPx5 Arabidopsis thaliana
 3058|HvGPx02| Hordeum vulgare
85
 5743|ZmGPx02| Zea mays
99
 2657|HvGPx06| Hordeum vulgare
 6242|MviGPx| Mesostigma viride
 CrGPxA
100
 2590|CreGPx02| Chlamydomonas reinhardtii
 CrGPxB
 3185|CreGPx06| Chlamydomonas reinhardtii
100
 PpGPxB
 CrGPxC
 2960|SobGPx01| Scenedesmus obliquus
69
 3074|CreGPx03| Chlamydomonas reinhardtii
89
 3184|CreGPx05| Chlamydomonas reinhardtii
96
0.20
0.15
0.10
0.05
0.00
0.35
0.30
0.25
